# Development of a High-Density Genetic Map Based on Specific Length Amplified Fragment Sequencing and Its Application in Quantitative Trait Loci Analysis for Yield-Related Traits in Cultivated Peanut

**DOI:** 10.3389/fpls.2018.00827

**Published:** 2018-06-26

**Authors:** Zhihui Wang, Dongxin Huai, Zhaohua Zhang, Ke Cheng, Yanping Kang, Liyun Wan, Liying Yan, Huifang Jiang, Yong Lei, Boshou Liao

**Affiliations:** Key Laboratory of Biology and Genetic Improvement of Oil Crops, Ministry of Agriculture, Oil Crops Research Institute of the Chinese Academy of Agricultural Sciences, Wuhan, China

**Keywords:** peanut, high-density genetic map, SLAF-seq, QTL analysis, yield

## Abstract

High-density genetic maps (HDGMs) are very useful for genomic studies and quantitative trait loci (QTL) mapping. However, the low frequency of DNA polymorphisms in peanut has limited the quantity of available markers and hindered the construction of a HDGM. This study generated a peanut genetic map with the highest number of high-quality SNPs based on specific locus amplified fragment sequencing (SLAF-seq) technology and a newly constructed RIL population (“ZH16” × “sd-H1”). The constructed HDGM included 3,630 SNP markers belonging to 2,636 bins on 20 linkage groups (LGs), and it covers 2,098.14 cM in length, with an average marker distance of 0.58 cM. This HDGM was applied for the following collinear comparison, scaffold anchoring and analysis of genomic characterization including recombination rates and segregation distortion in peanut. For QTL mapping of investigated 14 yield-related traits, a total of 62 QTLs were detected on 12 chromosomes across 3 environments, and the co-localization of QTLs was observed for these traits which were significantly correlated on phenotype. Two stable co-located QTLs for seed- and pod-related traits were significantly identified in the chromosomal end of B06 and B07, respectively. The construction of HDGM and QTL analysis for yield-related traits in this study provide useful information for fine mapping and functional analysis of genes as well as molecular marker-assisted breeding.

## Introduction

Peanut (*Arachis hypogaea* L., 2*n* = 20) is an economically important oilseed crop that is cultivated worldwide and it is one of the major grain legumes in tropical and subtropical regions. It is widely grown in over 100 countries, and has the global annual production of 42.4 Mt and area of 25.7 Mha in 2014 (http://faostat.fao.org/). However, increased global demand for production presents a challenge for peanut breeders to increase their yield. It is essential to conduct peanut breeding and genetic studies, such as linkage mapping or association analysis-based trait mapping, marker-assisted selection (MAS) breeding, and map-based gene cloning. For these studies, the density of the genetic map is very important because it provides a foundation for quantitative trait loci (QTL) mapping and further identification of genes of interest (Petroli et al., 2012; Song et al., 2012).

Great efforts have been made to construct peanut genetic maps using different types of molecular markers. Initial genetic maps were mainly developed based on the first generation molecular markers including random amplified polymorphic DNA (RAPD) (Hilu and Stalker, 1995), restriction fragment length polymorphism (RFLP) (Halward et al., 1993, 2011) and amplified fragment length polymorphism (AFLP) (He and Prakash, 1997; Tallury et al., 2005). These genetic maps commonly have low marker density with total marker numbers < 200. Subsequently, microsatellite markers have emerged as preferred DNA marker for conducting genetic and genomic studies in cultivated peanut. Varshney et al. (2009) published the first SSR-based genetic linkage map with 135 loci on 22 linkage groups spanning 1,271 cM. Later, Hong et al. (2010) mapped 175 SSR markers in 22 linkage groups developed from three cultivated crosses. Gautami et al. (2012) developed a consensus map with 293 SSR loci covering 2,840.8 cM based on two RIL populations. Qin et al. (2012) constructed two individual genetic maps with 236 and 172 marker loci, respectively, and then integrated them into a consensus map with 324 marker loci covering 1,352 cM genetic distance. Wang et al. (2012) constructed a genetic map with a total of 318 SSR markers covering 1,674.4 cM based on BAC-end sequences (BES). Shirasawa et al. (2012) created the high density SSR-based map of a single population of cultivated peanut, which generated 21 linkage groups covering 2,166.4 cM with 1,114 loci. Recently, Huang et al. (2016) constructed a high density linkage map with 1,219 SSR loci covering total map length of 2,038.75 cM.

Unfortunately, developing molecular markers in peanut has become labor-consuming and time-costing because cultivated peanut inherently has a very low frequency for DNA polymorphisms (Pandey et al., 2012; Varshney et al., 2013). For example, among nearly 10,000 SSR-based molecular markers, only 14.5% were polymorphic and 6.4% were mapped in peanut (Zhao et al., 2012). Therefore, the discovery of a sufficient number of molecular markers in cultivated peanuts is very challenging. SNP markers exhibit advantages in this case as they owned abundant DNA variations used for genetic markers (Brookes, 1999; Liao and Lee, 2010). Nagy et al. (2012) established a HDGM with 1,724 EST-SNP markers spanning 1,081.3 cM over 10 linkage groups in diploid species *A. duranensis*. Bertioli et al. (2014) used a 1,536 GoldenGate SNP assay in diploid and tetraploid RIL mapping populations, and constructed genetic maps containing 384 SNP markers in diploid and 772 SNP markers in tetraploid peanut. Using double-digest restriction-site-associated DNA sequencing (ddRAD-seq) technique and *de novo* SNP genotyping, we previously developed a SNP-based HDGM containing 1,685 SNPs covering 1,446.7 cM with an average distance of 0.86 cM between adjacent markers (Zhou et al., 2014). Nevertheless, efforts in identifying SNPs and constructing a HDGM in tetraploid peanut are limited due to few HDGMs existed in tetraploid peanut. The release of the draft genome sequence for both ancestral species of cultivated peanut, namely, *A. duranensis* (A genome) and *A. ipaensis* (B genome) in 2016 (Bertioli et al., 2016; Chen X. et al., 2016), has facilitated SNP discovery and genotyping.

Similar to restriction-site-associated DNA sequencing (RAD-seq) (Miller et al., 2007), ddRAD-seq (Peterson et al., 2012) and genotype-by-sequencing (GBS) (Poland et al., 2012), SLAF-seq, combined next-generation sequencing (NGS) with the use of restriction enzymes, are recently developed high-throughput methods for SNP marker discovery and genotyping (Sun et al., 2013). Using this technology, SNP markers have been widely applied for HDGM construction in various plants, such as sesame (Zhang et al., 2013), soybean (Qi et al., 2014), grape (Guo et al., 2014), Mei (Zhang et al., 2015b), cucumber (Zhu et al., 2016), and tetraploid cotton (Zhang et al., 2016). In this study, we successfully applied this technology to construct a HDGM with thousands of SNP markers in tetraploid peanut.

Yield is the most important and complex agronomical traits in crops. The yield traits include plant architecture and the pod- and seed-related traits (Holbrook and Stalker, 2003; Shirasawa et al., 2012). Selvaraj et al. (2009) identified five QTLs associated with differences between bulks for seed length, pod length, number of pods per plant, 100-seed weight. Fonceka et al. (2012) detected a total of 26 QTLs explaining 9.2–20.6% of the yield component traits. Shirasawa et al. (2012) identified a total of 23 significant QTLs explaining the phenotype variance ranged from 4.8 to 28.2% for the 15 investigated traits including pod- and seed-traits. Huang et al. (2015) detected 24 QTLs and each QTL explained 1.69–18.70% of the phenotypic variance for 10 yield traits. Chen W. et al. (2016) identified 39 QTLs explaining 1.25–26.11% of the phenotypic variations for pod length and width and seed length. For these yield-related QTLs, many of them explained minor or moderate phenotypic variation (Salas et al., 2006; Xu et al., 2011; Niu et al., 2013; Kato et al., 2014), and revealed the underlying complex genetic basis in peanut and other crops (Zuo and Li, 2014; Wang et al., 2015; Peng et al., 2016). However, compared to the comprehensive QTL studies on rice, oilseed rape and soybean (Varshney et al., 2010, 2013; Pandey et al., 2014), our understanding of the genetic basis for these traits in peanut are limited.

In the present study, a recombinant inbred line (RIL) population consisting of 242 individuals was derived from the cross between two tetraploid peanut genotypes, “ZH16” and “sd-H1.” The female parent, “ZH16” (*A. hypogaea var. vulgaris*), is a cultivar with large-seeded with pink testa and two seeds per pod. The male parent, “sd-H1” (*A. hypogaea var. fastigiata*), is a germplasm with small-seeded with red testa and three seeds in each pod. The yield-related traits were significantly different between two parents and extensive transgressive segregation in RIL population was observed, which are suitable for QTL mapping based on molecular markers. Here, genotype data were generated and SNP markers were discovered by SLAF-seq of the parents and RILs, and a HDGM of peanut was successfully constructed. To test the utility of this map, QTL mapping of 14 yield-related traits was conducted across 3 environments.

## Materials and methods

### Plant material and trait phenotyping

A F_6_ RIL population including 242 lines was developed from a cross between homozygous cultivars “ZH16” and “sd-H1.” Field experiments including the parents and segregating populations in years 2015 (Wuchang, E 114° 34′/N 30° 59′) and 2016 (Wuchang, E 114° 34′/N 30° 59′ and Yangluo, E 114° 52′/N 30° 59′) were conducted at Oil Crops Research Institute of the Chinese Academy of Agricultural Sciences, Hubei Province, China. The seeds were sown at the end of April of each experimental year. Each accession was planted in a single row, with 8 ± 10 plants in each row and 10-cm intervals between plants within each row, and 30-cm intervals between rows. The parental genotypes were also sown after every 50 rows as the controls. Randomized blocks were arranged, and average 10 individuals for each line in each block were selected for phenotype investigationfor PH, TBN, PL, PW, LWRP, SL, SW, LWRS, HPW, and HSW. The length and width of the two-seeded pods and seeds were measured using a parallel rule. The pods and seeds were also weighed on a digital scale. The PL/PW and SL/SW were calculated using PL and PW and SL and SW.

### Statistical analysis of broad-sense heritability

Broad-sense heritability was calculated using the equation *h*^2^ = σ_g_^2^/(σ_g_^2^ +σ_ge_^2^/n +σ_e_^2^/nr). In the equation, σ_g_^2^, σ_ge_^2^, and σ_e_^2^ were defined as the genetic variance, the interaction variance between genotype and environment, and the residual error variance, respectively. The n and r in the equation were defined as the number of environments and the number of replications, respectively. The general linear model (GLM) was used to estimate each variance component by SAS software. The Pearson's correlation coefficient was calculated for pair-wise comparison matrix of traits across the environments.

### Library construction and high-throughput sequencing

The genomic DNA was extracted using young leaves from each accession by a modified CTAB method. Through scanning the restriction endounclease sites on reference genome, the HaeIII endonuclease was chosen to digest the genomic DNA. The details of the SLAF-seq strategy and library construction were previously described (Sun et al., 2013; Zhang et al., 2015b). Finally, DNA fragments belonging to 400–530 bp in size were excised and diluted for paired-end sequencing which was performed on an Illumina HiSeq 2500 system (Illumina, Inc., San Diego, CA, USA). All SLAF-seq raw data from this study have been submitted to the NCBI SRA database (http://www.ncbi.nlm.nih.gov/sra/) under accession no. SRP108694.

### Grouping and genotyping of sequence data

The same procedure for read clustering and SNP genotyping was applied according to Sun et al. (2013) and Zhang et al. (2015b). After discarding the low-quality reads, the remaining reads were mapped to the reference genome by BWA software (Li and Durbin, 2009). The two progenitor genome sequences of tetraploid peanut were concatenated as the reference genome for read mapping (Bertioli et al., 2016). Only uniquely mapped reads were considered to identify SLAF markers (SLAFs). The SLAFs with less than four alleles were considered as polymorphic and potential markers. The Bayesian approach was used for genotype scoring, and the markers with >30% of missing data were further filtered out before genetic map construction, as described previously (Zhang et al., 2015b).

### Linkage map construction

The procedure used for linkage map construction was the same as Zhang et al. (2015b), including ordering of markers by HighMap strategy (Zhang et al., 2015b), error correction by SMOOTH strategy (van Os et al., 2005), and imputation of missing genotypes by k-nearest neighbor algorithm (Huang et al., 2012). The multipoint method of maximum likelihood was applied for adding the skewed markers to the genetic map (Xu and Hu, 2009). The genetic distance between markers was calculated using the Kosambi mapping function, and also compared with the marker orders of each group obtained by MSTMap (Wu et al., 2008). For inconsistent regions of marker orders, we used more rigorous parameters (distance < 20 cM between adjacent markers and *P*-value < 10^−5^) to re-calculate and adjust them. A bin is defined as a position on the genetic map containing a clustering of markers showing a genetic distance of zero between each other.

### QTL analysis using HDGM

The QTLs were detected using the composite interval mapping method in Windows QTL Cartographer 2.5 (Silva Lda et al., 2012). The permutation test was repeated 1000 times using 1.0 cM step and 5 control markers, and the LOD threshold (*P* < 0.05) were obtained for declaring significant QTLs. QTLs were considered as effective when the LOD scores were between 3.0 and the LOD threshold from the permutation test. Positive and negative additive effects mean that the favorable alleles were derived from parent “ZH16” and “sd-H1,” respectively. The QTL meta-analysis was performed to integrate the QTLs from different environments and traits using BioMeractor 4.2 software (Sosnowski et al., 2012).

## Results

### SLAF sequencing and genotyping

According to the results of pilot experiment, the HaeIII enzyme was selected for SLAF library construction. Considering the low rate of DNA polymorphisms in peanut, we sequenced a high amount of data of the parents and the RIL population to obtain a sufficient number of markers. A total of 524.83 Gb of data containing 2,624.07 M paired-end reads of 100 bp in length were obtained. The Q30 ratio was 90.40% and GC content was 43.25% in average. Of these high-quality data (Supplementary Table S1), 12.60 Gb were from the male parent “sd-H1” with 62,997,078 reads, and 11.59 Gb were from the female parent “ZH16” with 57,928,805 reads. Read numbers for the F6 population ranged from 6,800,354 to 18,885,615 with an average of 10,343,589. The number of SLAFs in male and female parents was 737,780 and 732,877, corresponding to 49,660,470 and 45,483,812 reads, respectively. The average sequencing depth for each SLAF marker was 67.31- and 62.06-fold in male and female parents, respectively. In the F6 population (Supplementary Table S1), the number of SLAFs ranged from 489,875 to 680,491 with an average of 589,904, and the coverage ranged from 9.04 to 27.92-fold with an average of 13.75-fold.

After filtering repetitive SLAFs, 824,866 SLAFs were detected, and 7.6% of these were polymorphic (Table 1). According to the genotype encoding rule, we obtained the number of SLAFs corresponding to eight segregation patterns (ab × cd, ef × eg, hk × hk, lm × ll, nn × np, aa × bb, ab × cc, and cc × ab) (Supplementary Figure S1). 28,720 polymorphic SLAFs containing 31,526 SNPs belonging to aa × bb segregation pattern were used in the following construction of linkage map because the two parents used for the cross are homozygous lines with a genotype of aa or bb. Finally, the low-quality SLAFs were discarded when they are with a parental sequence depth of < 10 × , completeness < 70%, and significant segregation distortion (*P* < 0.001).

**Table 1:** SLAF-seq data summary for peanut F_6_ population.

**Total reads**	
No. of reads (M)	2,624.07
Reads in high-quality SLAFs (M)	2,051.92
Reads in repeat SLAFs (M)	133.21
Reads in low depth SLAFs (M)	281.48
**HIGH-QUALITY SLAFS**	
No. of SLAFs	824,866
Average depth in parents	64.685
Average depth in individuals	13.76
**POLYMORPHIC SLAFS**	
No. of polymorphic SLAFs	63,026
Average depth in parents	123.71
Average depth in individuals	20.61
**HIGH-QUALITY SLAF MARKERS**	
No. of high-quality SLAF markers	3,630

*M, million; No, number; SLAF, specific length amplified fragment*.

### High-density genetic map construction

**Figure 1:**
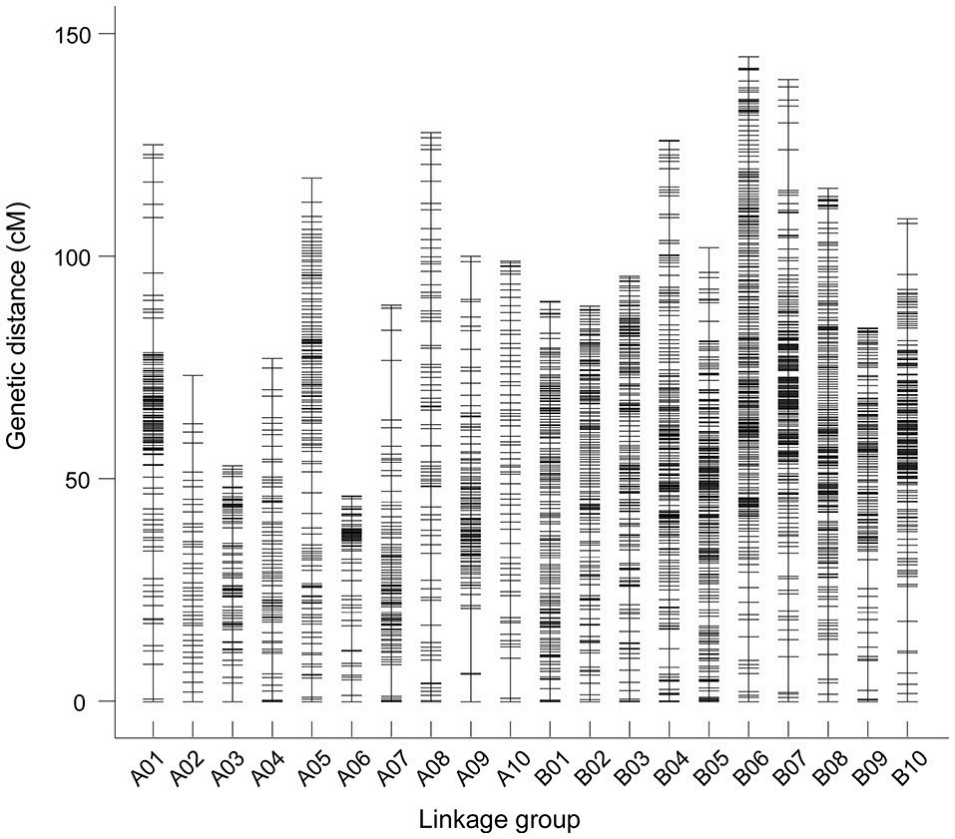
The markers distributed on 20 linkage groups of peanut. Each marker was indicated using a black bar. The x-axis and y-axis represent the number of linkage groups and the genetic distance, respectively.

The final map included 3,630 markers belonging to 2,636 bins on the 20 linkage groups (LGs) (Figure 1 and Supplementary Figure S2) and was 2,098.14 cM in length with an average inter-marker distance of 0.58 cM. As shown in Table 2, B07 was the largest LG which has 429 markers, with the length of 144.64 cM and an average distance of 0.34 cM. On the contrary, A02 was the smallest LG which has only 43 markers, with the length of 77.58 cM and an average distance of 1.80 cM. The linkage degree between the markers was reflected by “Gap ≤ 5,” which ranged between 93.15 and 100%, and showed an average value of 98.34%. The largest gap on this map was 15.44 cM, which was located on A09, followed by 14.10 cM on A07.

**Table 2:** Basic characteristics of peanut linkage groups.

**Linkage goups**	**Markers**	**Bins**	**Total distance (cM)**	**Average distance (cM)**	**Largest gap (cM)**	**Gaps ≤ 5 (%)**	**Segregation distorted markers**	**SDR number**	**Recombination rates (cM/Mb)**
A01	175	126	132.44	0.76	13.12	95.18	0	0	1.90
A02	43	43	77.58	1.80	11.34	95.12	37	4	4.04
A03	178	74	54.10	0.30	4.45	100.00	0	0	1.04
A04	82	78	84.02	1.02	5.11	97.40	0	0	2.26
A05	162	131	120.55	0.74	5.68	99.37	0	0	3.06
A06	126	52	47.17	0.37	5.74	99.17	116	2	1.86
A07	101	90	93.35	0.92	14.10	96.00	4	0	2.17
A08	77	76	140.02	1.82	6.36	93.15	41	2	4.75
A09	150	106	106.05	0.71	15.44	97.99	2	0	2.32
A10	66	65	102.27	1.55	9.52	98.44	1	0	2.75
B01	190	151	85.27	0.45	3.87	100.00	0	0	1.62
B02	174	144	95.28	0.55	3.39	100.00	132	7	2.78
B03	219	157	100.20	0.46	4.35	100.00	0	0	1.99
B04	245	183	133.52	0.54	5.47	99.59	4	0	3.15
B05	285	193	105.06	0.37	5.84	99.65	3	0	1.88
B06	267	265	153.42	0.57	5.33	99.62	22	2	1.60
B07	429	195	144.64	0.34	9.68	99.06	29	0	2.93
B08	211	208	123.46	0.59	5.89	99.50	1	0	2.22
B09	130	128	83.73	0.64	7.08	98.44	48	1	2.27
B10	320	171	116.02	0.36	11.81	99.05	248	2	1.87
Total	3630	2636	2098.14	0.58	/	98.34	688	20	2.42

There are three different types of markers in genetic map including 3,519 “SNP_only,” 101 “InDel_only,” and 10 “SNP&InDel” markers, accounting for 96.94, 2.78, and 0.28%, respectively (Supplementary Figure S3). Among the 3,519 markers of the “SNP_only” type, 95.28% had a single SNP locus and the others had two or three SNP loci. Two transition types of SNPs, including Y (T/C) and R (A/G), accounted for 36.05 and 35.05% of all markers, respectively. The other four transversion types of SNPs, including S (G/C), M (A/C), K (G/T), and W (A/T), ranged from 3.48 to 11.15% of all of the SNPs (Supplementary Figure S3). To confirm the authenticity of the identified SNPs, 19 out of 20 randomly selected SNPs were confirmed by Sanger sequencing (Supplementary Table S2).

Among the 3,630 markers, chi-square testing revealed that 688 (19%) showed significant segregation distortion, with 337 (49%) favoring the elite “ZH16” allele and 351 (51%) favoring the germplasm sd-H1 allele (Table 2). Segregation distortion regions (SDRs) were defined when at least four skewed markers were clustered. We found 20 SDRs including 688 distorted segregation markers on 7 chromosomes as follows: A02 (37), A06 (116), A08 (41), B02 (132), B06 (22), B09 (48), and B10 (248) (Supplementary Figure S4).

### The collinearity and evaluation of the genetic map

**Figure 2:**
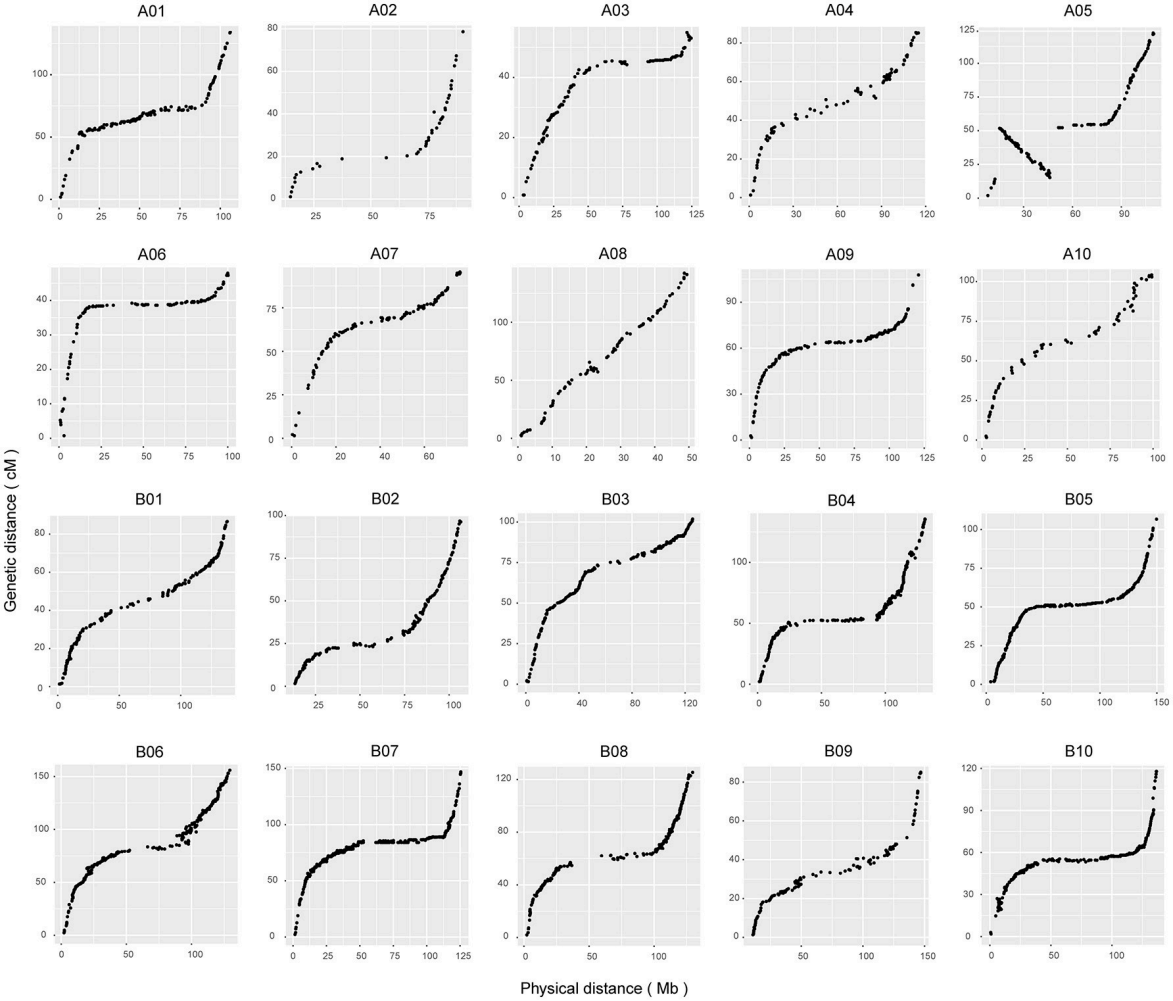
Collinearity analyses of all of the linkage groups with genome sequences. The x-axis scales the physical positions of markers based on reference sequences. The y-axis represents the genetic distance of the markers in centimorgans accordingly.

To assess the quality of this genetic map, a haplotype map was generated for each individual of the F_6_ population (Supplementary Figure S5), which reflects the double crossover and the recombination events (West et al., 2006). We further conducted a comparison between the genetic and physical positions of the markers based on the reference genome. A high collinearity between them indicated that the markers were placed accurately within each LG (Figure 2). A large inversed segment on chromosome A05 (15–45 cM) was found, which was in opposite orientation between genetic and physical positions (Figure 2). Subsequently, the recombination rates along chromosomes were calculated by comparing the genetic distance to the physical distance (Mb) (Supplementary Figure S6). It varied among different chromosomes, ranging from 1.04 centimorgans per megabase (cM/Mb) for chromosome A03 to 4.75 cM/Mb for chromosome A08, and a genome-wide average recombination rate of 2.42 cM/Mb. In addition, we anchored 62 scaffolds accounting for 5.8 Mb of the total length to specific chromosomal positions using genetic markers on scaffolds, which allowed updating of the genome assembly (Supplementary Table S3).

### Yield-related phenotypic traits of the parents and individuals

**Figure 3:**
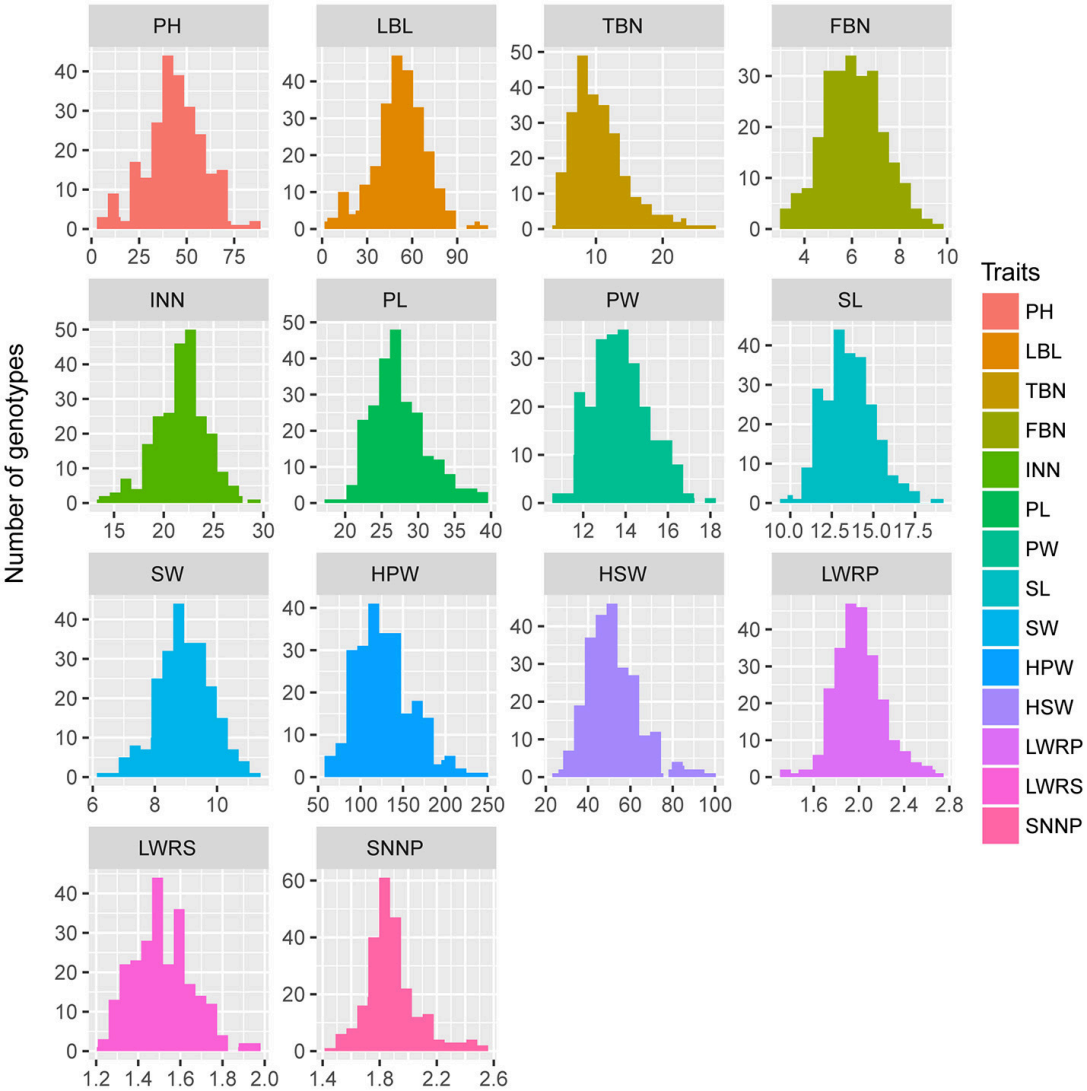
Phenotypic distributions of 14 yield-related traits in the RIL population.

Parent “ZH16” is a high-yield Spanish type cultivar with larger pods and seed size and two seeds in each pod, whereas parent “sd-H1” is a low-yield Valencia type cultivar with smaller pods and seeds and three seeds in each pod. The two parents showed significant differences (*P* < 0.001) in 14 yield-related traits, which were evaluated in 3 different environments in RILs (Table 3). The traits examined showed approximately the same phenotypic data distribution for all 3 environments (Table 3). A continuous distribution were shown for all traits in RIL population (Figure 3), indicating that these traits were controlled by multiple genes and belonged to a quantitative inheritance pattern.

**Table 3:** Phenotypic variation of “ZH16,” “sd-H1,” and RILs grown in three different environments.

**Trait**	**WuH15**			**WuH16**			**YangL16**			***h*^2^**
	**ZH16**	**sd-H1**	**RIL**	**ZH16**	**sd-H1**	**RIL**	**ZH16**	**sd-H1**	**RIL**	
**PLANT ARCHITECTURE**										
Plant height (cm)	34.80	16.14	41.06 ± 16.37	36.13	27.21	43.83 ± 15.93	33.55	22.63	45.83 ± 15.29	0.85
Lateral branch length (cm)	40.10	19.57	50.38 ± 19.52	39.40	35.10	51.4 ± 18.26	37.85	27.35	54.09 ± 18	0.86
Total branch number	13.70	9.14	9.08 ± 3.26	9.98	6.36	10.84 ± 5.34	10.53	6.63	11.13 ± 4.98	0.69
Fruiting branch number	9.60	5.71	6.47 ± 1.75	8.07	4.29	5.81 ± 1.37	6.78	4.13	5.86 ± 1.59	0.66
Internode number	18.30	21.43	23.43 ± 3.67	15.10	20.13	20.3 ± 2.99	15.98	19.70	21.22 ± 2.92	0.72
**POD AND SEED**										
Pod length (mm)	33.8	24.0	28.65 ± 5.32	30.5	29.5	27.2 ± 4.42	29.3	25.3	26.74 ± 4.14	0.77
Pod width (mm)	17.2	12.0	14.36 ± 2.78	16.6	11.7	13.76 ± 1.48	15.9	11.5	13.3 ± 1.42	0.75
Seed length (mm)	19.2	11.8	13.94 ± 1.71	16.4	11.9	13.5 ± 1.68	16.3	11.7	13.2 ± 1.54	0.74
Seed width (mm)	14.0	7.3	9.62 ± 6.15	11.1	7.6	9.01 ± 0.85	11.1	7.0	8.61 ± 0.78	0.67
Hundred-pod weight (g)	257.36	106.80	133.01 ± 35.76	200.34	112.03	128.7 ± 38.96	217.55	93.76	123.75 ± 36.87	0.78
Hundred-seed weight (g)	112.19	34.80	57.20 ± 23.47	79.72	30.88	51.38 ± 13.22	83.46	28.44	47.58 ± 11.67	0.75
Length-width ratio of pod	1.97	2.00	2.02 ± 0.30	1.84	2.50	1.97 ± 0.23	1.85	2.22	2.01 ± 0.24	0.74
Length-width ratio of seed	1.37	1.62	1.48 ± 0.16	1.50	1.57	1.51 ± 0.15	1.48	1.69	1.55 ± 0.15	0.81
Seed number per pod	1.84	2.66	1.98 ± 0.25	1.85	2.55	1.86 ± 0.21	1.83	2.26	1.83 ± 0.23	0.75

**Figure 4:**
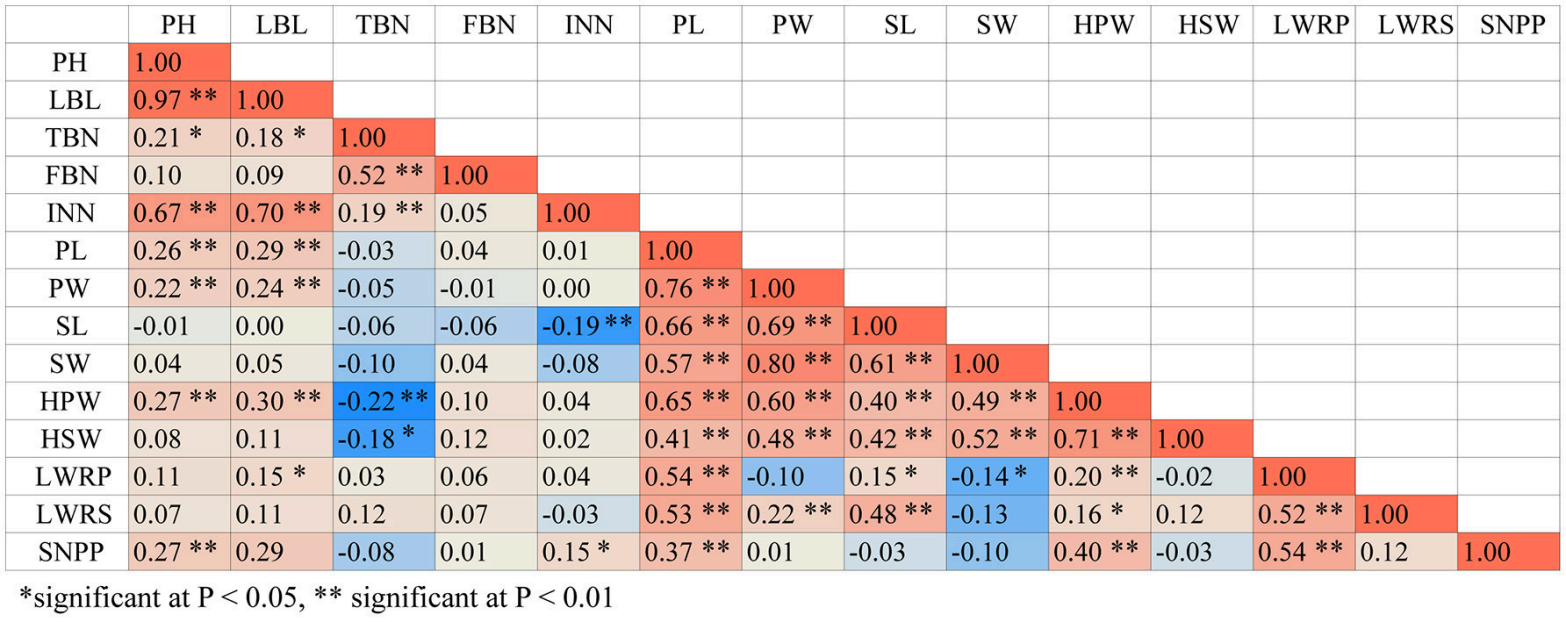
The correlation coefficients between pair-wise trait matrix. The phenotypic values averaged from three environments were used for Pearson's correlation test.

We conducted ANOVA analysis for all 14 traits across the environments, and calculated the effects of genotype (G), environment (E), and genotype-environment interactions (G × E) on the traits (Table 4). They showed high broad-sense heritability, ranging from 65.7 to 86.2%, suggesting a major role of genetic factors in the expression of these traits as well as a considerable proportion of environmental variation affecting these traits. To elucidate their relationships, correlation coefficients (r) between the traits were calculated across the various environments (Figure 4). Plant height (PH) and lateral branch length (LBL) were strongly positively correlated, with a correlation coefficient of 0.97. Significant positive relationships also have been found for internode number (IN) and lateral branch length (LBL) (*r* = 0.70), Pod length (PL) and pod width (PW) (*r* = 0.76), PW and seed width (SW) (*r* = 0.80), hundred-pod weight (HPW) and hundred-seed weight (HSW) (*r* = 0.71). The significant phenotypic correlations among these traits coincided with the results of QTL co-localization (see next).

**Table 4:** The broad-sense heritability for the 14 yield-related traits of RILs.

**Traits**	**Source**	**DF**	**Sum of square**	**Mean square**	***F* value**	***P***	***h^2^***
PH	Genotype	241	222022.25	921.25	8.29	< 0.0001	0.85
	Environment	2	5637.91	2818.95	25.37	< 0.0001	
	Genotype × Environment	482	65655.57	136.21	0.75	0.007	
	Error	707	78542.32	111.09			
LBL	Genotype	241	292737.35	1214.68	7.35	< 0.0001	0.86
	Environment	2	7685.70	3842.85	23.24	< 0.0001	
	Genotype × Environment	482	80913.96	167.87	1.02	0.4265	
	Error	701	115917.26	165.36			
TBN	Genotype	241	17561.85	72.87	4.94	< 0.0001	0.69
	Environment	2	1372.36	686.18	46.49	< 0.0001	
	Genotype × Environment	482	10846.26	22.50	1.52	< 0.0001	
	Error	702	10360.24	14.76			
FBN	Genotype	241	1691.50	7.02	2.98	< 0.0001	0.66
	Environment	2	44.17	22.08	9.38	< 0.0001	
	Genotype × Environment	481	1155.22	2.40	1.02	0.4049	
	Error	711	1674.39	2.35			
INN	Genotype	241	7113.49	29.52	4.10	< 0.0001	0.72
	Environment	2	2488.78	1244.39	172.67	< 0.0001	
	Genotype × Environment	482	3930.74	8.16	1.13	0.0681	
	Error	708	5102.31	7.21			
PL	Genotype	241	14956.85	62.06	5.19	< 0.0001	0.77
	Environment	2	694.35	347.18	29.02	< 0.0001	
	Genotype × Environment	482	6948.13	14.42	1.20	0.0126	
	Error	696	8327.75	11.97			
PW	Genotype	241	2152.07	8.93	3.48	< 0.0001	0.75
	Environment	2	199.89	99.94	38.99	< 0.0001	
	Genotype × Environment	482	1080.32	2.24	0.87	0.944	
	Error	697	1786.62	2.56			
SL	Genotype	241	2115.09	8.78	4.88	< 0.0001	0.74
	Environment	2	189.03	94.52	52.54	< 0.0001	
	Genotype × Environment	481	1093.49	2.27	1.26	0.0026	
	Error	674	1212.51	1.80			
SW	Genotype	241	622.76	2.58	2.76	< 0.0001	0.67
	Environment	2	126.26	63.13	67.41	< 0.0001	
	Genotype × Environment	482	416.03	0.86	0.92	0.8321	
	Error	684	640.56	0.94			
HPW	Genotype	241	1148055.56	4763.72	5.94	< 0.0001	0.78
	Environment	2	25146.29	12573.15	15.68	< 0.0001	
	Genotype × Environment	482	494384.01	1025.69	1.28	0.0015	
	Error	696	558193.77	802.00			
HSW	Genotype	241	147186.52	610.73	3.40	< 0.0001	0.75
	Environment	2	16738.02	8369.01	46.65	< 0.0001	
	Genotype × Environment	482	74534.50	154.64	0.86	0.9599	
	Error	680	121984.72	179.39			
LWRP	Genotype	241	45.38	0.19	4.90	< 0.0001	0.74
	Environment	2	0.51	0.25	6.61	0.0014	
	Genotype × Environment	482	23.82	0.05	1.29	0.0013	
	Error	696	26.77	0.04			
LWRS	Genotype	241	19.58	0.08	6.69	< 0.0001	0.81
	Environment	2	0.79	0.40	32.74	< 0.0001	
	Genotype × Environment	481	7.53	0.02	1.29	0.0012	
	Error	673	8.17	0.01			
SNNP	Genotype	241	40.29	0.17	4.83	< 0.0001	0.75
	Environment	2	7.61	3.80	109.89	< 0.0001	
	Genotype × Environment	482	20.45	0.04	1.23	0.0073	
	Error	700	24.24	0.03			

### QTL analysis and candidate gene identification for agronomic and yield-related traits

**Figure 5:**
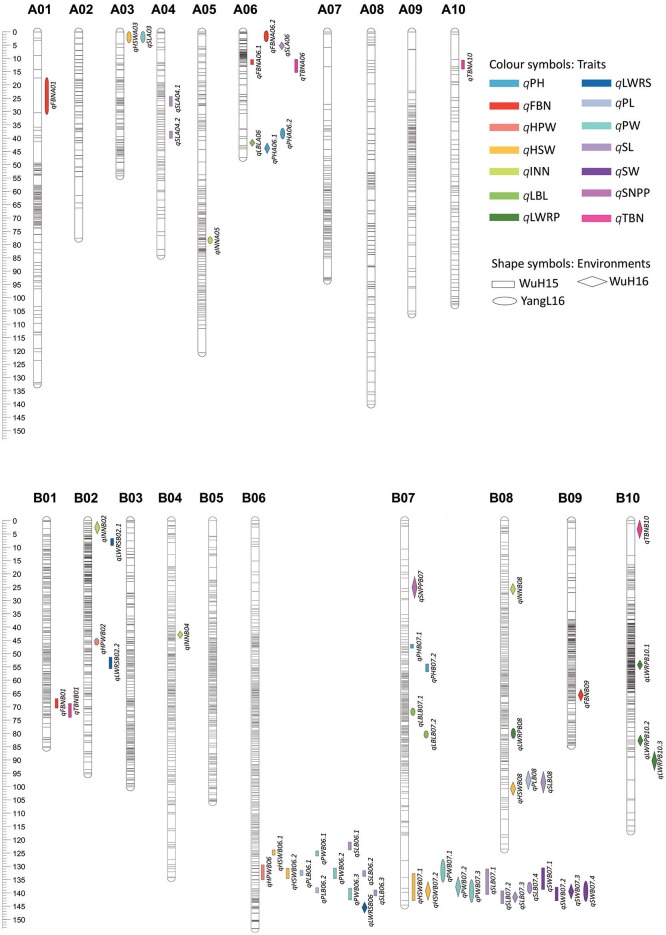
The distribution of significant QTLs on chromosomes.

A total of 62 QTLs for all of the 14 traits were detected on 12 chromosomes across the 3 environments (“WuH15,” “WuH16,” and “YangL16”), and explained 4.03 to 18.9% of the observed phenotypic variation, respectively (Supplementary Table S4). Furthermore, 33 consensus QTLs were obtained by the trait-by-trait meta-analysis. For the five plant architecture traits, We have detected five QTLs for PH, three QTLs for LBL, four QTLs for total branch number (TBN), five QTLs for fruiting branch number (FBN) and four QTLs for INN, most QTLs for these traits were located on A01, A06, A10, B01, B06, B07, and B10. It is showed that several QTLs for different traits were co-localized, such as one region (41.1–46.0 cM) on A06 for traits PH (*qPHA06.1* from “WuH16”) and LBL (*qLBLA06* from “WuH16”), one region on A06 (10.2–14.4 cM) for traits FBN (*qFBNA06.1* from “WuH15”) and TBN (*qTBNA06* from “WuH15”), and one region on B01 (67.5–74.0 cM) for traits FBN (*qFBNB01* from “WuH15”) and TBN (*qTBNB01* from “WuH15”), respectively.For the pod- and seed-related traits, we have identified three QTLs for PL, six QTLs for PW, four QTLs for length-width ratio of pod (LWRP), six QTLs for HSW, two QTLs for HPW and thirteen QTLs for SL, their identified QTLs were localized to A04, B02, B06, B07, and B08, and co-localized to two genomic regions: B06 (121.5–141.0 cM) and B07 (131.2–142.9 cM) (Figure 5), including *qPLB06.1–2, qPWB06.1–3, qSLB06.1–3, qHPWB06, qHSWB06.1-2* from “WuH15”, *qPWB07.1–3* from “WuH16” and “YangL16,” qHSWB07.1–2 from “WuH15” and “WuH16”, *qSLB07.1–4* and *qSWB07.1–4* from “WuH15,” “WuH16” and “YangL16.” These results showed that the pleiotropic QTLs contained multiple tightly linked genes for different traits or the gene that affects multiple traits (Hall et al., 2006).

All these annotated genes were identified underlying predicted QTLs and then blasted to find homologous genes with functional descriptions in *A. thaliana* (Supplementary Table S5). We conducted GO analysis of these genes, and found that many of these distributed in GO categories, such as “GO:0032502 developmental process,” “GO:0010926 anatomical structure formation,” “GO:0040007 growth,” “GO:0065007 biological regulation,” “GO:0030528 transcription regulator activity” (Supplementary Figure S7). We further checked the functional annotation and description of genes within two obviously co-located QTLs of seed- and pod-related traits in B06 and B07 since these two QTL regions were also confirmed by our following Bulk segregant analysis (BSA) of the trait of interest (Unpublished data). There are 63 and 76 candidate genes in the two regions, respectively, and some candidate genes were found to be associated with yield-related traits based on previous studies. In the pleiotropic QTL on B06, two genes, *Araip.10014506* and *Araip.10014509*, were homologous to *AT1G08840* and *AT1G80260*, respectively, which were reported to be related to embryo development (McElver et al., 2001; Jia et al., 2016). In the pleiotropic QTL on B07, the *Araip.10036332 and Araip.10036333*, a pair of tandemly duplicated genes, were homologous to *aap8* (AMINO ACID PERMEASE 8, *AT1G10010*). This gene in *A. thaliana* plays an important role in the regulation of carbon metabolism and transport by sink strength (Santiago and Tegeder, 2016, 2017). Carbon fixation and assimilation as well as sucrose partitioning to siliques were strongly decreased when *app8* mutant plants transitioned to the reproductive phase. Based on these results, these candidate genes within QTLs should be further investigated, including gene cloning and functional analysis.

## Discussion

Cultivated peanut, an allotetraploid (2n = 4x = 40), is a self-pollinated crop with a large genome (~ 2.8 Gb). Investigations on the genetic diversity of peanut germplasm resources have indicated that cultivated peanut possesses an extremely narrow genetic base (Jiang et al., 2010; Ren et al., 2010; Belamkar et al., 2011; Wang et al., 2011; Mukri et al., 2012; Upadhyaya et al., 2012), which is likely due to its monophyletic origin from a single hybridization event between two ancient diploid species (Burow et al., 2009). The low polymorphism rates have been revealed for a variety of markers in peanut including 6.6% for RAPD (Subramanian et al., 2000), 3.6% for AFLP (He and Prakash, 1997), 10.4% for EST-SSR (Liang et al., 2009), 14.5% for SSR (Zhao et al., 2012), 7.6% for SNPs in this study. Low levels of polymorphisms limit the quantity of available markers and hinder the construction of a HDGM. However, the development of next-generation sequencing has facilitated in obtaining thousands of SNPs in the peanut genome and constructing a HDGM. Using ddRAD-seq technology, we previously reported a HDGM for cultivated peanut that comprises 1,267 bins and 1,685 SNPs, covering 1,446.7 cM with an average distance of 0.86 cM between adjacent markers (Zhou et al., 2014). In this study, we developed a new HDGM using SLAF-seq technology, which includes 2,597 bins and 3,568 markers, spanning 2,098.1 cM with an average inter-marker distance of 0.59 cM. To our knowledge, this genetic map has the highest number of SNPs for cultivated peanut to date.

The success in constructing HDGM is mainly attributed to the mature pipeline on sequencing and analyzing technology of SLAF-seq, the high sequencing depth of the parents (60-fold) and RILs (20-fold), the relatively large population size, and more importantly, the availability of a reference genome. Compared to the *de novo* method used by Zhou et al. (2014), the reference genome used in this study guarantees the accuracy of mapping, clustering and genotyping for SLAF markers, as well as serves as a tool for the validation of the quality of the genetic map. A highly conserved genome between diploid and tetraploid species of *Arachis* was revealed by the good collinearity between the genetic and physical map, which may be due to its short evolutionary history after two diploid genomes merged approximately 3,500 years ago (Gary Kochert et al., 1996). On the other hand, the constructed HDGM also could help in determining the genomic distribution of segregation distortion and recombination rates. In this study, we identified 20 SDRs on 7 chromosomes where the genetic selection factors for gametophyte competition probably existed. Further studies investigating the segregation distortion on specific regions can elucidate the mechanism underlying the distorted loci. Furthermore, information on the chromosomal distribution of recombination events will aid in defining the centromere regions as well as the recombination hot and cold spots on various chromosomes.

The constructed HDGM was used to identify QTLs for yield-related traits. Several QTLs explaining moderate phenotypic variation were identified, which is in agreement with the results of previous studies on other crops in which yield-related traits were always controlled by multiple small-effect QTLs (Peiffer et al., 2014; Zhang et al., 2015a). Some identified QTLs in this study were located on the same chromosomes as previously reported for PH, and TBN (Fonceka et al., 2012; Huang et al., 2015), and pod and seed traits (Fonceka et al., 2012; Shirasawa et al., 2012; Pandey et al., 2014; Huang et al., 2015, 2016). Because no markers were shared between our map and these reported maps, we were unable to compare QTL positions among the different maps. However, the known and novel QTLs for yield-related traits should both be detected in our materials, as the genetic basis of these traits was mainly dependent on peanut genotypes. In addition, although the broad-sense heritability was relatively higher in these traits, the stability and accuracy of QTLs are still affected by environmental factors, including the season and climatic conditions. Nevertheless, we detected several stable QTLs that are common across different years and environments as well as several pleiotropic QTLs. The co-localization of QTLs was observed for yield-related traits in this study, which is similar to that in other crops, such as soybean (Xie et al., 2014), rapeseed (Shi et al., 2009; Li et al., 2014; Liu et al., 2015), and rice (Zuo and Li, 2014). The significant pleiotropic QTLs suggest that these traits are influenced by several genes that control different aspects of complex metabolic pathways, and they might have resulted from the artificial selection and rapid evolution of multiple traits in peanut breeding (Yoshizawa et al., 2013). In addition, several genes are associated with yield traits in stable and pleiotropic QTLs, and these play important roles in seed development, sugar transport, and transcriptional regulation. The identified candidate genes in pleiotropic QTLs provide information on the genetic basis of these traits, thereby facilitating the selection of varieties during molecular breeding. Further transcriptomic and gene-directed studies for these candidate genes may facilitate the elucidation of the molecular mechanisms underlying yield-related traits.

## Author contributions

ZW, YL, and BL conceived and designed the experiments, ZW, DH, ZZ, KC, LW, and LY performed the agronomic traits measurements of the plant materials, ZW, analyzed the data and wrote the manuscript, ZW, HJ, YL, and BL revised the manuscript. All authors have read and approved the final version of the manuscript.

### Conflict of interest statement

The authors declare that the research was conducted in the absence of any commercial or financial relationships that could be construed as a potential conflict of interest.

## Abbreviations

AFLP: amplified fragment length polymorphism
ddRAD-seq: double-digest restriction-site associated DNA sequencing
EST: expressed sequence tag
FBN: Fruiting branch number
GBS: genotype-by-sequencing
HDGM: high-density genetic map
HPW: Hundred-pod weight
HSW: Hundred-seed weight
IN: Internode number
LBL: Lateral branch length
LG: linkage group
LWRP: Length-width ratio of pod
LWRS: Length-width ratio of seed
NGS: next-generation sequencing
PH: Plant height
PL: Pod length
PW: Pod width
QTL: quantitative trait loci
RAPD: random amplified polymorphic DNA
RFLP: restriction fragment length polymorphism
RIL: recombinant inbred line
SDR: segregation distortion region
SLAF-seq: specific locus amplified fragment sequencing
SL: Seed length
SNP: single nucleotide polymorphism
SNPP: Seed number per pod
SSR: simple sequence repeat
SW: Seed width
TBN: Total branch number.
