# Supplementary material for: Development of a High-Density Genetic Map Based on Specific Length Amplified Fragment Sequencing and Its Application in Quantitative Trait Loci Analysis for Yield-Related Traits in Cultivated Peanut
Source: Front Plant Sci. 2018 Jun 26;9:827. doi: 10.3389/fpls.2018.00827 (PMC6028809; doi:10.3389/fpls.2018.00827)
Supplement: Supplementary file 1 [file Table_1.PDF]

Supplementary Table S1. The SLAF-seq data of the parents and RILs.

SampleID	Total Reads	Total Bases （Gb）	GC Percent	Q30 Percenta	all.marker.num.depth.stat		
					MarkerNum	Total_Depth	Aver_Depth
ZH16	57,928,805	11.59	43.15	90.35	732877	45483812	62.06
sd-H1	62,997,078	12.60	42.70	90.12	737780	49660470	67.31
RIL001	9,576,973	1.92	42.95	90.71	563677	7606270	13.49
RIL002	9,573,357	1.91	43.20	91.16	549129	8469701	15.42
RIL003	9,718,822	1.94	43.13	91.00	554371	8206654	14.80
RIL004	10,096,042	2.02	43.72	89.12	549868	8364612	15.21
RIL005	9,391,540	1.88	44.10	90.86	585336	6792033	11.60
RIL006	9,268,172	1.85	43.06	90.45	521600	7018959	13.46
RIL007	9,804,916	1.96	43.10	90.09	606348	8166177	13.47
RIL008	12,126,758	2.43	43.50	89.20	600901	7367775	12.26
RIL009	11,179,673	2.24	43.20	90.11	569930	7309968	12.83
RIL010	10,715,284	2.14	43.26	91.39	614490	7329506	11.93
RIL011	10,032,524	2.01	43.06	91.56	602273	7950262	13.20
RIL012	11,367,145	2.27	42.75	90.48	596862	8333520	13.96
RIL013	11,297,937	2.26	42.64	90.03	585866	7929660	13.53
RIL014	8,346,761	1.67	42.96	90.44	614555	8109888	13.20
RIL015	10,652,815	2.13	42.91	90.78	589171	8236734	13.98
RIL016	9,620,307	1.92	43.10	90.65	586120	8275899	14.12
RIL017	12,886,060	2.58	42.54	90.83	602642	8132484	13.49
RIL018	11,775,655	2.36	43.52	90.59	591287	9391419	15.88
RIL019	9,192,044	1.84	43.38	90.85	574032	8623432	15.02
RIL020	11,250,520	2.25	43.48	90.54	598942	9578666	15.99
RIL021	8,865,394	1.77	44.03	89.49	602227	6979627	11.59
RIL022	12,008,610	2.40	43.84	89.15	552323	8957533	16.22
RIL023	11,262,755	2.25	43.11	90.82	579502	8022217	13.84
RIL024	9,899,462	1.98	43.69	90.17	586195	8973410	15.31
RIL025	9,495,430	1.90	43.47	90.80	616542	8955418	14.53
RIL026	9,433,763	1.89	43.44	90.67	560027	6967656	12.44
RIL027	11,381,098	2.28	42.86	90.98	520657	6794419	13.05
RIL028	10,448,893	2.09	43.50	91.02	576422	7676794	13.32
RIL029	10,085,036	2.02	43.44	90.92	581168	8476833	14.59
RIL030	9,683,051	1.94	43.83	90.53	574726	8122248	14.13
RIL031	9,049,520	1.81	43.17	90.83	570012	10614888	18.62
RIL032	12,647,423	2.53	44.35	89.54	592441	7990577	13.49
RIL033	12,239,513	2.45	43.62	90.17	546579	6595660	12.07
RIL034	11,755,651	2.35	44.41	90.58	528377	10410320	19.70
RIL035	12,571,618	2.51	44.04	90.57	564866	8938487	15.82
RIL036	7,550,466	1.51	43.78	91.10	609604	7549642	12.38
RIL037	13,469,225	2.69	43.92	89.65	489875	9757037	19.92
RIL038	6,828,883	1.37	43.07	89.37	579584	9889582	17.06
RIL039	6,961,154	1.39	43.10	89.64	600786	7766353	12.93
RIL040	8,405,385	1.68	43.42	89.83	559749	10680772	19.08
RIL041	7,864,118	1.57	43.13	89.50	558958	8532862	15.27
RIL042	10,175,312	2.04	43.27	89.24	594599	9329710	15.69

RIL043	10,272,721	2.05	43.57	90.46	595937	10079882	16.91
RIL044	8,361,473	1.67	42.59	90.99	660037	8616579	13.05
RIL045	11,383,634	2.28	43.37	90.06	611772	7038308	11.50
RIL046	11,152,804	2.23	43.35	91.22	576404	6550804	11.36
RIL047	11,353,805	2.27	43.88	91.24	580089	6841148	11.79
RIL048	12,912,720	2.58	43.65	91.08	592209	7149744	12.07
RIL049	12,903,348	2.58	43.25	90.99	522161	10398438	19.91
RIL050	12,078,686	2.42	43.74	90.82	522203	9124999	17.47
RIL051	11,523,104	2.30	43.07	90.91	562205	10182640	18.11
RIL052	8,889,437	1.78	43.18	91.16	585204	8357565	14.28
RIL053	7,945,349	1.59	43.71	88.80	552941	10197165	18.44
RIL054	11,298,180	2.26	43.97	89.06	549602	7632081	13.89
RIL055	12,152,967	2.43	44.45	88.25	614401	8266094	13.45
RIL056	12,088,158	2.42	43.78	89.96	644715	6208678	9.63
RIL057	7,908,335	1.58	43.56	90.47	571942	8409920	14.70
RIL058	8,695,048	1.74	42.70	90.75	568408	8362307	14.71
RIL059	7,599,258	1.52	43.67	90.39	593485	6755690	11.38
RIL060	13,146,903	2.63	44.15	89.82	528929	8416370	15.91
RIL061	8,561,734	1.71	43.79	90.73	516678	10816733	20.94
RIL062	9,226,079	1.85	43.35	90.88	544249	8751999	16.08
RIL063	9,013,578	1.80	43.18	90.49	636421	9106662	14.31
RIL064	7,834,998	1.57	43.44	91.26	533909	7359158	13.78
RIL065	7,729,918	1.55	43.70	90.49	556816	7707052	13.84
RIL066	8,747,088	1.75	43.41	90.44	529099	10485608	19.82
RIL067	8,918,835	1.78	43.70	90.68	530658	6764057	12.75
RIL068	10,298,768	2.06	43.04	90.67	540805	7564434	13.99
RIL069	8,961,369	1.79	43.64	90.38	498691	8786211	17.62
RIL070	9,812,908	1.96	43.08	90.28	606276	9045058	14.92
RIL071	8,541,176	1.71	42.84	91.40	607495	6782892	11.17
RIL072	7,518,203	1.50	42.58	91.02	577788	8876150	15.36
RIL073	7,977,391	1.60	42.71	91.42	551393	10777535	19.55
RIL074	9,175,063	1.84	43.15	88.74	573787	9025216	15.73
RIL075	8,726,413	1.75	43.19	89.74	584040	5660404	9.69
RIL076	11,047,204	2.21	43.72	88.38	670182	9535051	14.23
RIL077	11,006,079	2.20	43.50	88.99	579717	7426608	12.81
RIL078	10,030,048	2.01	44.07	89.35	630335	9726497	15.43
RIL079	8,030,224	1.61	42.77	89.60	573946	10211865	17.79
RIL080	8,621,429	1.72	43.02	89.76	562943	9422454	16.74
RIL081	8,179,873	1.64	43.38	89.65	566955	11399045	20.11
RIL082	7,509,069	1.50	42.93	89.46	552932	9713298	17.57
RIL083	7,117,078	1.42	43.58	90.03	541907	9759631	18.01
RIL084	6,800,354	1.36	42.67	90.01	597257	7615014	12.75
RIL085	10,372,264	2.07	43.69	89.16	514158	10545304	20.51
RIL086	8,959,022	1.79	42.60	90.08	560675	8745244	15.60
RIL087	9,736,258	1.95	43.11	90.68	541399	10214708	18.87
RIL088	13,315,627	2.66	43.27	89.66	569633	8691552	15.26

RIL089	10,165,369	2.03	42.73	90.29	580091	8359072	14.41
RIL090	11,365,013	2.27	43.23	90.14	573698	9208695	16.05
RIL091	9,458,955	1.89	43.47	90.67	602050	9705855	16.12
RIL092	10,171,427	2.03	42.98	90.31	571435	7140240	12.50
RIL093	10,504,664	2.10	42.71	90.44	613212	7801810	12.72
RIL094	10,426,951	2.09	42.96	90.44	578039	6829841	11.82
RIL095	9,241,842	1.85	42.66	90.18	535833	14958968	27.92
RIL096	11,600,958	2.32	43.62	89.93	582938	10169092	17.44
RIL097	10,670,574	2.13	42.55	90.62	568264	9373887	16.50
RIL098	11,287,453	2.26	42.41	90.35	569288	7716828	13.56
RIL099	9,773,535	1.95	42.67	90.27	598117	7862401	13.15
RIL100	10,397,013	2.08	42.74	90.62	595873	9125869	15.32
RIL101	10,539,523	2.11	42.83	91.52	618029	7191457	11.64
RIL102	8,789,937	1.76	43.82	90.80	602523	5726744	9.50
RIL103	8,887,334	1.78	43.05	90.71	561724	7591613	13.51
RIL104	10,377,103	2.08	43.21	91.13	610934	7724102	12.64
RIL105	9,463,591	1.89	43.40	91.02	603279	8414808	13.95
RIL106	9,295,332	1.86	43.03	90.91	605027	8882328	14.68
RIL107	9,335,398	1.87	43.20	91.36	586064	8854421	15.11
RIL108	10,123,350	2.02	43.33	91.19	623962	8556445	13.71
RIL109	10,612,541	2.12	43.32	91.58	625204	6579492	10.52
RIL110	10,273,992	2.05	43.06	91.03	551557	7903824	14.33
RIL111	10,549,890	2.11	43.36	89.24	589809	9075437	15.39
RIL112	10,592,520	2.12	43.40	90.64	623827	7541280	12.09
RIL113	10,381,412	2.08	42.99	91.32	604488	8741765	14.46
RIL114	11,994,189	2.40	43.31	90.80	616889	6791470	11.01
RIL115	11,014,004	2.20	43.24	90.50	608177	8452876	13.90
RIL116	12,223,048	2.44	43.35	90.89	596689	8039463	13.47
RIL117	8,851,354	1.77	42.99	91.40	615556	8279737	13.45
RIL118	11,321,428	2.26	42.82	90.77	612326	7685506	12.55
RIL119	10,155,849	2.03	42.93	90.59	592595	6087543	10.27
RIL120	11,546,636	2.31	43.54	90.99	573099	6459980	11.27
RIL121	8,913,492	1.78	43.08	90.92	616804	7379000	11.96
RIL122	8,805,965	1.76	43.63	90.75	672438	9383339	13.95
RIL123	9,821,179	1.96	43.20	90.47	678534	9342867	13.77
RIL124	10,706,342	2.14	42.90	90.76	600684	6858787	11.42
RIL125	10,394,693	2.08	42.99	89.57	623867	7844321	12.57
RIL126	13,400,590	2.68	42.64	89.06	611332	6942067	11.36
RIL127	10,274,644	2.05	43.23	89.43	584102	6263527	10.72
RIL128	8,488,849	1.70	43.18	89.19	603311	8137804	13.49
RIL129	13,287,083	2.66	43.11	89.51	628075	7713659	12.28
RIL130	9,711,476	1.94	43.25	89.83	605012	6837443	11.30
RIL131	12,561,195	2.51	43.27	89.17	599474	7356780	12.27
RIL132	12,660,618	2.53	42.52	90.00	675036	9549258	14.15
RIL133	9,951,204	1.99	42.85	90.28	599121	5843284	9.75
RIL134	13,733,857	2.75	43.01	89.97	595061	7213928	12.12

RIL135	10,725,386	2.15	42.63	90.15	666610	9246433	13.87
RIL136	11,938,716	2.39	43.12	90.24	584461	7984487	13.66
RIL137	12,749,956	2.55	42.73	90.25	658879	8506894	12.91
RIL138	11,250,890	2.25	43.74	90.38	610701	6800398	11.14
RIL139	9,072,716	1.81	42.93	90.64	571484	5906086	10.33
RIL140	8,666,960	1.73	43.03	90.77	608457	6787237	11.15
RIL141	9,326,829	1.87	43.65	90.02	508553	5672188	11.15
RIL142	13,356,291	2.67	42.76	90.30	549849	6718390	12.22
RIL143	11,554,486	2.31	42.61	90.25	580762	7474441	12.87
RIL144	12,870,643	2.57	42.53	91.05	532005	9859386	18.53
RIL145	10,798,100	2.16	43.34	90.36	610695	8865428	14.52
RIL146	13,015,596	2.60	42.50	90.74	548538	8861230	16.15
RIL147	9,829,888	1.97	43.52	90.57	607808	8735559	14.37
RIL148	10,534,761	2.11	43.00	90.25	584761	8469791	14.48
RIL149	7,940,087	1.59	43.31	90.70	557708	7912898	14.19
RIL150	10,561,505	2.11	42.90	90.48	608148	9966293	16.39
RIL151	8,666,230	1.73	43.23	90.71	552489	9816874	17.77
RIL152	10,745,265	2.15	42.73	90.84	617292	5580065	9.04
RIL153	13,700,196	2.74	42.61	90.66	585203	8904707	15.22
RIL154	11,190,269	2.24	43.16	91.07	629994	7371339	11.70
RIL155	11,796,733	2.36	43.56	90.47	581579	6564038	11.29
RIL156	9,403,779	1.88	43.22	90.72	610704	7294975	11.95
RIL157	9,894,005	1.98	43.26	90.34	632961	7698000	12.16
RIL158	13,465,918	2.69	42.95	90.20	576792	10162503	17.62
RIL159	8,669,215	1.73	43.14	90.11	620249	7466841	12.04
RIL160	11,209,990	2.24	42.99	90.28	632923	7732357	12.22
RIL161	11,472,677	2.29	42.80	90.73	623631	7413115	11.89
RIL162	8,762,769	1.75	43.65	91.10	587144	7363819	12.54
RIL163	11,394,996	2.28	43.48	90.56	590709	9025989	15.28
RIL164	13,591,870	2.72	42.81	90.72	613299	8170176	13.32
RIL165	11,425,520	2.29	43.08	90.66	608664	7895716	12.97
RIL166	7,389,509	1.48	44.03	91.14	600488	7675379	12.78
RIL167	12,457,580	2.49	44.07	90.96	607798	7501042	12.34
RIL168	9,422,837	1.88	42.92	90.59	615212	7142675	11.61
RIL169	12,319,877	2.46	42.94	90.84	644514	9501643	14.74
RIL170	12,091,882	2.42	43.20	90.69	669909	9451167	14.11
RIL171	14,321,714	2.86	42.65	90.31	676128	8961259	13.25
RIL172	12,286,498	2.46	42.75	90.66	676118	9680094	14.32
RIL173	12,327,157	2.47	42.73	90.27	564729	5889190	10.43
RIL174	9,749,464	1.95	43.20	91.11	667613	10340634	15.49
RIL175	13,280,807	2.66	42.98	90.72	540667	5329470	9.86
RIL176	11,272,903	2.25	43.77	90.59	556148	5474869	9.84
RIL177	12,767,084	2.55	42.24	90.77	658427	7802749	11.85
RIL178	11,067,207	2.21	43.16	90.86	595672	6545040	10.99
RIL179	10,734,756	2.15	43.66	90.85	573519	6185094	10.78
RIL180	12,465,849	2.49	43.63	90.49	662669	7914163	11.94

RIL181	9,090,336	1.82	43.34	91.04	593022	8009566	13.51
RIL182	9,889,046	1.98	43.08	91.19	546916	6694057	12.24
RIL183	8,821,458	1.76	43.79	90.60	642502	8883598	13.83
RIL184	18,885,615	3.78	42.94	90.89	617697	8768085	14.19
RIL185	13,077,565	2.62	43.47	90.74	623433	8815941	14.14
RIL186	12,268,597	2.45	42.43	90.84	647306	10003128	15.45
RIL187	9,773,662	1.95	42.90	90.83	636125	10187837	16.02
RIL188	10,081,045	2.02	43.63	90.96	621071	7203058	11.60
RIL189	11,579,484	2.32	42.66	90.68	620074	9422485	15.20
RIL190	7,436,384	1.49	44.13	91.25	594001	9124062	15.36
RIL191	9,581,493	1.92	42.98	91.27	581431	7052717	12.13
RIL192	9,984,432	2.00	43.96	90.96	568095	6172563	10.87
RIL193	10,758,524	2.15	43.24	90.84	634686	8606334	13.56
RIL194	11,397,373	2.28	43.28	90.90	655429	9187550	14.02
RIL195	11,227,256	2.25	42.88	91.19	649019	9315658	14.35
RIL196	10,904,676	2.18	43.26	91.39	565065	6173621	10.93
RIL197	8,426,852	1.69	43.70	91.54	597936	6538371	10.93
RIL198	9,933,376	1.99	42.61	91.12	566769	5915380	10.44
RIL199	11,672,034	2.33	43.36	90.98	565227	7275033	12.87
RIL200	8,821,286	1.76	43.78	90.84	674092	10003859	14.84
RIL201	10,854,983	2.17	43.13	90.80	591853	6646565	11.23
RIL202	10,333,818	2.07	43.39	90.67	563057	7276946	12.92
RIL203	10,644,088	2.13	43.54	89.46	590370	7126272	12.07
RIL204	9,945,881	1.99	43.57	89.79	596169	6169492	10.35
RIL205	7,886,656	1.58	43.72	89.28	569830	6051490	10.62
RIL206	8,349,905	1.67	43.65	89.18	595555	6781858	11.39
RIL207	9,377,261	1.88	43.02	89.50	589505	6986705	11.85
RIL208	12,129,599	2.43	43.63	89.29	563183	8128824	14.43
RIL209	12,052,131	2.41	43.33	89.78	591748	7012749	11.85
RIL210	10,090,582	2.02	43.92	90.53	596077	7719851	12.95
RIL211	8,812,673	1.76	43.40	90.56	585694	7752287	13.24
RIL212	7,947,864	1.59	43.50	91.24	545967	6752865	12.37
RIL213	10,296,693	2.06	43.11	90.49	555075	5971067	10.76
RIL214	9,817,537	1.96	43.09	91.05	590516	6305591	10.68
RIL215	8,788,328	1.76	42.91	89.51	592816	7195237	12.14
RIL216	9,490,674	1.90	43.74	89.64	560466	6903620	12.32
RIL217	12,486,313	2.50	44.11	89.38	639085	8499716	13.30
RIL218	7,475,486	1.50	43.28	89.61	648452	8590614	13.25
RIL219	9,140,988	1.83	43.41	90.91	667578	7677143	11.50
RIL220	10,214,601	2.04	42.97	90.09	565230	6295674	11.14
RIL221	11,118,094	2.22	43.83	88.75	651235	9342736	14.35
RIL222	8,697,539	1.74	43.44	89.71	576227	6828134	11.85
RIL223	7,596,052	1.52	43.56	89.80	564363	6404606	11.35
RIL224	8,728,402	1.75	43.27	89.66	524712	5944528	11.33
RIL225	7,301,310	1.46	43.23	90.25	564554	5525623	9.79
RIL226	8,643,552	1.73	43.16	90.62	527825	5423465	10.28

RIL227	9,380,239	1.88	42.59	90.77	633105	7959553	12.57
RIL228	12,524,474	2.50	42.89	90.43	554772	7149203	12.89
RIL229	13,281,844	2.66	43.16	90.27	585076	7668435	13.11
RIL230	11,266,814	2.25	43.84	90.56	658597	10429541	15.84
RIL231	10,832,054	2.17	42.81	89.69	558034	8100650	14.52
RIL232	10,006,032	2.00	43.04	89.94	580540	8780734	15.13
RIL233	12,613,247	2.52	42.71	90.21	590539	8935327	15.13
RIL234	12,406,076	2.48	42.74	89.65	614268	7420407	12.08
RIL235	7,140,226	1.43	43.40	90.46	571606	8026693	14.04
RIL236	11,255,633	2.25	42.82	90.27	584306	8358101	14.30
RIL237	9,481,097	1.90	43.63	90.07	585065	8276986	14.15
RIL238	8,477,360	1.70	43.82	89.49	556628	7320440	13.15
RIL239	9,425,376	1.89	43.92	90.14	680491	9004121	13.23
RIL240	12,824,457	2.56	42.94	90.01	612516	8492448	13.86
RIL241	9,512,163	1.90	43.31	90.12	519003	9007919	17.36
RIL242	9,860,163	1.97	43.43	91.17	533731	7774069	14.57
Offspring_Aver	10,343,589	2	43	90	589904	8085834	13.71
