# Supplementary material for: Development of a High-Density Genetic Map Based on Specific Length Amplified Fragment Sequencing and Its Application in Quantitative Trait Loci Analysis for Yield-Related Traits in Cultivated Peanut
Source: Front Plant Sci. 2018 Jun 26;9:827. doi: 10.3389/fpls.2018.00827 (PMC6028809; doi:10.3389/fpls.2018.00827)
Supplement: Supplementary file 2 [file Table_2.PDF]

Supplementary Table S2. SNP markers selected for PCR validation and Sanger sequencing.

**Marker name**	**Chr.**	Primer 5' sequence	Primer 3' sequence	Type	Start	End	PCR Product length	Confirm by Sanger
AhSNP10012475	B04	ATAGAGGGAACGCCGGATAC	CGCTGGTACCGATGACTTCT	W(A/T)	111577828	1.12E+08	449	Yes
AhSNP10111289	B04	AAAAGGCCAAAACCATGTGA	AACCGCAACTTCAACGACTC	R(A/G)	122764542	1.23E+08	644	Yes
AhSNP10153807	B04	TCCATTCGCATCTTCCTCTC	GGTCGGGTTGGTAAGGATCT	K(G/T)	123444664	1.23E+08	582	Yes
AhSNP10155468	B04	GCCATTTTGGTGGAGAGAGA	TGAAAGCATGCCAATGAGAG	Y(C/T)	94687223	94687497	593	Yes
AhSNP11477698	A09	TCCTGAGACCCACACCTTTC	TAGCATCTGCTGGGAGAACC	R(A/G)	4421740	4422000	266	Yes
AhSNP11545308	A01	ATAATTGCCGCTTCGAGTTG	TCTCATTCAGCACCCCTCTC	R(A/G)	45637624	45637838	631	Yes
AhSNP1100798	A03	GCCAAAGAGAGCATCCAAAA	GCCTGAAACCAAACATCCAC	K(G/T)	119454739	1.19E+08	220	Yes
AhSNP11211163	A09	TCCCCATGACCAAAAAGAAA	CTTTTTGCAGGTGCTTCCTC	R(A/G)	112692673	1.13E+08	349	Yes
AhSNP10726666	A09	GGATTTTGGCTTTGGGTTTT	ATCGCGTCGTTACTCGTTTC	R(A/G)	7876594	7876814	504	Yes
AhSNP10765552	A09	CTTGGCTAGGACCGTTGAAA	AGTTCGGCCCATTTAAGACC	W(A/T)	120344362	1.2E+08	495	Yes
AhSNP10893509	A09	ACCGATTTGAAGTTGGGTCA	ACATTCTGCCCATTGCATTT	R(A/G)	2136326	2136554	560	Yes
AhSNP1140112	A03	GGCACGCCTGTAACCTAAGA	CCGACCCTTTGACATTCATC	K(G/T)	46603238	46603416	745	Yes
AhSNP11535638	A01	GGACGTCTCCCAGACAGAAA	TGTGGCTAGGAACGGTCTTC	M(A/C)	26564902	26565064	599	Yes
AhSNP6894495	A06	GGGAAACACGAGGTTTGATG	GGGAGATTTCTGCGACAAGA	R(A/G)	17349098	17349191	94	Yes
AhSNP12687658	A10	ACTCGTTGGTCTGCCAACTC	GGATGACTGCTCGTCGATCT	Y(C/T)	88494416	88494731	316	Yes
AhSNP14010978	B02	TCTGGCGCTATTGCATGATA	CCACATCCACACCATTGAGA	K(G/T)	18197621	18197920	300	Yes
AhSNP1100798	A03	GCCAAAGAGAGCATCCAAAA	GCCTGAAACCAAACATCCAC	K(G/T)	119454739	1.19E+08	220	Yes
AhSNP15312345	B06	CAATCCTCTTCATGCGTGTG	TGGAACTCGCTCCCTAATTG	-	22460890	22461146	257	Yes
AhSNP10755076	A09	GGCACACAGCAGTACCCTCT	CCTCCCAAGTGCCCTTTTAT	R(A/G)	90454954	90455189	295	Yes
AhSNP10927678	A09	GTTTAATGCCGCTCCGAGTA	GCCTCCTCATCTCGAAACAA	M(A/C)	23481096	23481329	559	No
