# Supplementary material for: Development of a High-Density Genetic Map Based on Specific Length Amplified Fragment Sequencing and Its Application in Quantitative Trait Loci Analysis for Yield-Related Traits in Cultivated Peanut
Source: Front Plant Sci. 2018 Jun 26;9:827. doi: 10.3389/fpls.2018.00827 (PMC6028809; doi:10.3389/fpls.2018.00827)
Supplement: Supplementary file 3 [file Table_3.PDF]

Supplementary Table S3. The information of scaffolds anchored to chromosomes by SNP markers on the genetic map.

Marker on scaffold	Scaffold containing marker	scaffold length (bp)	Linkage group of marker on scaffold	cM position of marker on scaffold	The scaffold anchored to chromosome	
					the above marker position on chr (marker : start_end)	the next marker position on chr (marker : start_end)
AhSNP185714	Adur1240	122674	A01	57.697	AhSNP12099476:32614005_32614154	AhSNP12097817_29312006_29312184
AhSNP82373	Adur1348	94725	A01	62.621	AhSNP11997358:47827266_47827465	AhSNP11862058_49356546_49356702
AhSNP29547	Adur2662	11213	A01	66.68	AhSNP11953854:55913462_55913642	AhSNP12094937_53928273_53928440
AhSNP130992	Adur1889	24395	A01	68.699	AhSNP11622476:57304473_57304697	AhSNP11794151_58935273_58935431
AhSNP297910	Adur791	329380	A01	69.613	AhSNP11870163:67966290_67966573	AhSNP12034615_76397370_76397614
AhSNP155505	Adur1967	21068	A01	72.16	AhSNP11839888:80989374_80989501	AhSNP11809825_82073646_82073873
AhSNP212726	Adur1733	33765	A01	72.165	AhSNP11839888:80989374_80989501	AhSNP11809825_82073646_82073873
AhSNP265662	Adur1967	21068	A01	72.914	AhSNP11992346:78626979_78627231	AhSNP12129182_88423176_88423355
AhSNP243036	Adur2073	18695	A01	126.602	AhSNP12049024:104099175_104099382	AhSNP11937647_105801617_105801775
AhSNP243497	Adur944	239715	A02	12.764	AhSNP4818193:19099099_19099309	AhSNP4936288_24381750_24382002
AhSNP277970	Adur2435	12639	A02	24.888	AhSNP4898132:74214981_74215055	AhSNP5143826_74287597_74287814
AhSNP201960	Adur2096	17778	A03	31.415	AhSNP1786578:31967234_31967444	AhSNP1101369_32923611_32923751
AhSNP100131	Adur1326	98212	A03	42.749	AhSNP1253401:50600937_50601206	AhSNP1700031_56130911_56131158
AhSNP180941	Adur1560_1	5672	A03	44.427	AhSNP1753619:62366646_62366891	AhSNP1115470_66763751_66763913
AhSNP251630	Adur952	235461	A04	37.87	AhSNP13308355:21669520_21669746	AhSNP12990849_32183699_32183962
AhSNP228649	Adur731	364079	A04	44.959	AhSNP13204523:41068860_41069020	AhSNP13451511_52508258_52508499
AhSNP22887	Adur793	329235	A04	57.266	AhSNP13478895:82179777_82179777	AhSNP13580935_90573272_90573452
AhSNP180919	Adur2730	10956	A04	62.534	AhSNP13543831:93258209_93258496	AhSNP13186320_95147698_95147897
AhSNP234819	Adur2058	19148	A04	64.97	AhIndel13657684:99648444_99648666	AhSNP13365660_96323365_96323593
AhSNP39931	Adur995_2	47441	A05	67.632	AhSNP2044731:79442348_79442441	AhSNP2074856_80344454_80344616
AhSNP4441	Adur2102	17594	A05	68.264	AhSNP2087105:60398169_60398367	AhSNP2034790_65347740_65347876
AhSNP50520	Adur2425	12714	A05	68.264	AhSNP2087105:60398169_60398367	AhSNP2034790_65347740_65347876
AhSNP15379	Adur536	557318	A06	8.362	AhSNP6707621:79512312_79512505	AhSNP6690945_79430309_79430468
AhSNP230743	Adur159_2	45890	A06	8.652	AhSNP7058025:79435874_79436012	AhSNP6544774_71996646_71996912
AhSNP64605	Adur1582	49353	A06	9.112	AhSNP6675205:45614502_45614733	AhSNP6599913_49514478_49514643
AhSNP204477	Adur2546	11936	A06	9.774	AhSNP6802988:21265545_21265841	AhSNP6817144_17387211_17387432
AhSNP28882	Aipa775	29848	A06	9.774	AhSNP6802988:21265545_21265841	AhSNP6817144_17387211_17387432
AhSNP206174	Adur1762	30997	A07	68.115	AhSNP7208568:49842734_49842950	AhSNP7520128_50753748_50753901

AhSNP49189	Adur720	369560	A08	54.063	AhSNP6383143:19615600_19615737	AhSNP6459875_19948024_19948303
AhSNP22440	Adur720	369560	A08	54.563	AhSNP6459875:19948024_19948303	AhSNP6455127_23126582_23126739
AhSNP4318	Adur720	369560	A08	56.256	AhSNP6527606:21884807_21884968	AhIndel6393153_20203423_20203668
AhSNP30788	Adur720	369560	A08	57.749	AhSNP6347456:22701632_22701888	AhSNP6478538_22050091_22050235
AhSNP137917	Adur1891	24291	A09	38.446	AhSNP11178174:92967956_92968125	AhSNP11379531_92695908_92696050
AhSNP183013	Adur793	329235	A10	13.906	AhSNP12789091:3808536_3808813	AhSNP12624916_4217833_4218006
AhSNP173477	Adur1430	76654	A10	58.269	AhSNP12908944:34284404_34284616	AhSNP12509271_35427937_35428143
AhSNP18576	Aipa322	265164	B01	66.01	AhSNP6011453:127240287_127240531	AhSNP5825753_128542620_128542809
AhSNP273256	Aipa437	64108	B03	71.975	AhSNP16506658:53389724_53389896	AhSNP15950307_70067366_70067551
AhSNP141677	Aipa791	26406	B04	53.249	AhSNP10007678:94746088_94746324	AhSNP10497633_93197679_93197938
AhSNP240642	Aipa1346	11063	B05	49.89	AhSNP9277944:69601132_69601397	AhSNP9167281_72766474_72766666
AhSNP152722	Aipa750	35377	B06	78.409	AhSNP14591461:51870595_51870818	AhSNP14888648_73929251_73929513
AhSNP37360	Aipa896	20814	B06	78.782	AhSNP14888648:73929251_73929513	AhSNP15118150_78813084_78813243
AhSNP136918	Aipa750	35377	B06	81.573	AhSNP14610103:65729644_65729799	AhSNP15515607_84413381_84413668
AhSNP235471	Aipa288_2	18886	B06	82.909	AhSNP15384562:90012483_90012757	AhSNP14570087_87912960_87913156
AhSNP37636	Aipa502	48855	B07	81.796	AhSNP7822266:49068086_49068296	AhSNP8271139_71197572_71197842
AhSNP44920	Aipa390	96912	B07	81.796	AhSNP8207170:74598524_74598685	AhSNP8174519_73505573_73505834
AhSNP91901	Adur1477	67418	B08	30.357	AhSNP3945080:8766939_8767134	AhSNP4596887_7582587_7582737
AhSNP191457	Aipa1371	10860	B08	52.152	AhSNP4133496:27304624_27304874	AhSNP3884224_32854231_32854395
AhSNP190318	Aipa1371	10860	B08	52.152	AhSNP3884224:32854231_32854395	AhSNP4113364_30392078_30392254
AhSNP20636	Aipa1409	10689	B08	57.149	AhSNP3753208:34993879_34994123	AhSNP4331606_71539276_71539543
AhSNP33983	Aipa426	76157	B08	57.357	AhSNP4331606:71539276_71539543	AhSNP4071236_68736739_68736962
AhSNP41962	Aipa1334	11170	B08	57.567	AhSNP4071236:68736739_68736962	AhSNP3727328_67859110_67859352
AhSNP58471	Aipa731	39188	B08	58.568	AhSNP4371388:76025313_76025551	AhSNP4641937_71446534_71446784
AhSNP39226	Aipa426	76157	B08	58.78	AhSNP4604104:74306857_74307045	AhSNP3713450_73629768_73629940
AhSNP28064	Aipa1409	10689	B08	59.784	AhSNP4375169:86247730_86247915	AhSNP4330707_59067581_59067870
AhSNP33912	Aipa815	24126	B08	60.284	AhSNP4330707:59067581_59067870	AhSNP4118350_65962220_65962417
AhSNP161935	Aipa1123	13530	B08	60.376	AhSNP4118350:65962220_65962417	AhSNP4490748_66235489_66235556
AhSNP68037	Aipa1300	11492	B08	64.277	AhSNP4513387:100605548_100605831	AhSNP4594487_101754586_101754796
AhSNP140847	Aipa819	23984	B09	17.595	AhSNP2593623:20952864_20953052	AhSNP3082569_23256706_23256900
AhSNP152325	Aipa1026	16344	B09	20.36	AhSNP2619318:27695555_27695713	AhSNP3075758_31064548_31064750
AhSNP182380	Aipa1150	13063	B10	47.893	AhSNP640751:31860587_31860828	AhSNP58311_31060499_31060643

AhSNP169547	Adur1669	40284	B10	53.133	AhSNP620196:76466431_76466598	AhSNP334349_50025266_50025412
AhSNP14230	Aipa1324	11228	B10	55.357	AhSNP163173:98870296_98870547	AhSNP214780_106028384_106028583
