# Supplementary material for: Development of a High-Density Genetic Map Based on Specific Length Amplified Fragment Sequencing and Its Application in Quantitative Trait Loci Analysis for Yield-Related Traits in Cultivated Peanut
Source: Front Plant Sci. 2018 Jun 26;9:827. doi: 10.3389/fpls.2018.00827 (PMC6028809; doi:10.3389/fpls.2018.00827)
Supplement: Supplementary file 4 [file Table_4.PDF]

Supplementary Table S4. QTLs controlling yield-related traits detected in different environments.

Trait	QTL	Chr.	Marker interval	Position(cM)	Confidence interval(cM)	Env.	Year	PVE%	Additive effect	LOD
PH	*qPHA06.1*	A06	AhSNP6568422-AhSNP6794628	43.31	42.8-46	WuH16	2016	5.07	-3.63	3.02
	qPHA06.2	A06	AhIndel6843531-AhSNP6568422	39.41	37.3-40.1	YangL16	2016	6.10	-3.82	3.80
	qPHB06	B06	AhSNP15357423-AhSNP14573470	42.91	42.3-43.4	WuH15	2015	7.03	4.57	4.34
	qPHB07.1	B07	AhSNP7791819-AhSNP7702639	49.31	48.6-49.5	WuH15	2015	4.30	2.49	2.53
	qPHB07.2	B07	AhSNP7779427-AhSNP8072443	56.51	54.7-57	WuH15	2015	6.04	3.10	3.59
LBL	*qLBLA06*	A06	AhSNP7140384-AhSNP6794628	42.71	41.1-46	WuH16	2016	4.45	-3.90	2.71
	qLBLB07.1	B07	AhSNP8202839-AhSNP7885526	71.51	71.3-71.8	YangL16	2016	4.47	3.84	2.56
	qLBLB07.2	B07	AhSNP8113916-AhIndel8431318	81.61	80.1-82	YangL16	2016	4.43	3.81	2.53
TBN	*qTBNA06*	A06	AhIndel7054692-AhSNP6850752	11.21	10.4-14.4	WuH15	2015	7.16	-1.84	4.38
	qTBNA10	A10	AhSNP12677062-AhSNP12927391	13.11	10.9-15	WuH15	2015	5.12	-0.84	3.19
	qTBNB01	B01	AhSNP6125629-AhSNP5781856	70.01	68.7-74	WuH15	2015	4.45	0.65	2.79
	qTBNB10	B10	AhSNP945679-AhSNP834777	0.01	0-6	WuH16	2016	6.57	-1.38	4.09
FBN	*qFBNA01*	A01	AhSNP12141017-AhSNP11610128	25.31	17.3-30.3	WuH16	2016	6.12	-0.35	2.85
	qFBNA06.1	A06	AhIndel7054692-AhSNP6850752	10.61	10.2-11.3	WuH15	2015	6.26	-0.79	3.73
	qFBNA06.2	A06	AhSNP6677580-AhSNP7159472	0.21	0-3.1	WuH16	2016	4.40	-0.30	2.73
	qFBNB01	B01	AhSNP5837940-AhSNP6093023	68.71	67.5-70.6	WuH15	2015	6.61	0.50	3.97
	qFBNB09	B09	AhSNP2644292-AhIndel2712399	66.21	64.6-67.9	WuH16	2016	4.32	-0.29	2.70
INN	*qINNA05*	A05	AhSNP2324857-AhSNP2139010	78.01	77.3-78.9	YangL16	2016	4.85	0.81	2.88
	qINNB02	B02	AhSNP14395471-AhSNP14388597	3.11	0.9-4.4	WuH16	2016	4.24	0.63	2.60
	qINNB04	B04	AhIndel10025925-AhSNP10598525	44.41	42.8-44.6	WuH16	2016	4.17	-0.62	2.56
	qINNB08	B08	AhSNP4532078-AhIndel4487699	25.81	24.8-27.9	WuH16	2016	4.66	-0.65	2.86
PL	*qPLB06.1*	B06	AhSNP15007648-AhSNP14973104	132.21	131.9-133.2	WuH15	2015	18.99	5.18	11.60
	qPLB06.2	B06	AhSNP15460609-AhSNP15112056	139.51	138.9-140	WuH15	2015	16.78	4.15	8.16
	qPLB08	B08	AhSNP3749179-AhSNP4485440	97.41	94-100.4	WuH16	2016	4.30	0.93	2.53
PW	*qPWB06.1*	B06	AhSNP14709329-AhSNP15163391	124.81	124.3-125.7	WuH15	2015	15.94	4.07	7.80
	qPWB06.2	B06	AhSNP15007648-AhSNP15057182	132.21	131.4-134.5	WuH15	2015	17.09	6.08	8.57
	qPWB06.3	B06	AhSNP14591706-AhSNP14894764	139.51	138.5-142.4	WuH15	2015	14.89	4.06	7.11
	qPWB07.1	B07	AhSNP8362338-AhSNP8048484	130.91	127.7-134.3	YangL16	2016	15.07	4.32	7.55
	qPWB07.2	B07	AhSNP8505759-AhSNP7755889	139.81	134.6-142.8	WuH16	2016	14.57	4.33	6.80
	qPWB07.3	B07	AhSNP8505759-AhSNP7755889	138.31	135.4-142.8	YangL16	2016	15.65	4.34	7.37
SL	*qSLA03*	A03	AhSNP1417767-AhSNP1217642	0.01	0-3.5	YangL16	2016	5.48	-0.36	3.34
	qSLA04.1	A04	AhSNP13558548-AhSNP13394128	25.91	24.9-27.8	WuH15	2015	4.03	-0.08	2.50
	qSLA04.2	A04	AhSNP13308355-AhSNP13357771	38.31	37.6-39.9	WuH15	2015	5.56	-0.11	3.49
	qSLA06	A06	AhSNP6974142-AhSNP6816914	4.51	4.3-5.7	WuH16	2016	4.84	-0.57	3.12
	qSLB06.1	B06	AhSNP14947934-AhSNP15443760	122.41	121.5-124	WuH15	2015	16.91	5.06	7.87
	qSLB06.2	B06	AhSNP15007648-AhSNP14973104	132.21	131.6-133.4	WuH15	2015	17.90	5.07	8.91
	qSLB06.3	B06	AhSNP14732062-AhSNP15202567	139.51	139.3-141	WuH15	2015	15.96	4.06	7.66
	qSLB07.1	B07	AhSNP8444006-AhSNP8048484	137.31	131.2-139.8	WuH15	2015	14.98	3.05	7.04
	qSLB07.2	B07	AhSNP8505759-AhSNP8338430	142.91	139.8-143.9	WuH15	2015	14.41	4.05	7.17
	qSLB07.3	B07	AhSNP8505759-AhSNP7755889	140.81	139.3-142.9	WuH16	2016	18.21	6.49	10.88
	qSLB07.4	B07	AhSNP8505759-AhSNP8048484	138.31	136.3-139.8	YangL16	2016	15.75	3.40	7.53
	qSLB08	B08	AhSNP3749179-AhSNP3709171	97.41	94.6-102.1	WuH16	2016	4.05	0.34	2.55
SW	*qSWB07.1*	B07	AhSNP8444006-AhSNP8048484	134.31	130.9-138.3	WuH15	2015	15.55	5.03	7.53
	qSWB07.2	B07	AhSNP8505759-AhSNP7755889	140.81	138.3-142.9	WuH15	2015	15.58	5.03	7.27
	qSWB07.3	B07	AhSNP8505759-AhSNP8048484	138.31	136-141.8	WuH16	2016	15.43	4.21	7.58
	qSWB07.4	B07	AhSNP8505759-AhSNP7755889	139.31	135.9-142.9	YangL16	2016	15.06	4.18	7.97
HPW	*qHPWB02*	B02	AhSNP13792358-AhSNP14028184	45.61	44.9-46	YangL16	2016	5.86	9.24	3.47
	qHPWB06	B06	AhSNP14871490-AhSNP15057182	133.21	129.8-134.6	WuH15	2015	14.46	6.19	7.22
HSW	*qHSWA03*	A03	AhSNP1417767-AhSNP1217642	0.01	0-3.3	YangL16	2016	5.17	-2.68	3.13
	qHSWB06.1	B06	AhSNP15443760-AhSNP15128876	124.81	124.3-125.7	WuH15	2015	17.95	5.00	8.75
	qHSWB06.2	B06	AhSNP15007648-AhSNP15057182	133.21	131.4-134.6	WuH15	2015	16.50	5.56	7.90
	qHSWB07.1	B07	AhSNP8505759-AhSNP7755889	136.31	133.9-142.9	WuH15	2015	15.92	4.88	7.54
	qHSWB07.2	B07	AhSNP8505759-AhSNP7755889	138.31	136.5-142.8	WuH16	2016	16.72	3.58	7.81
	qHSWB08	B08	AhSNP4204715-AhSNP3996377	99.11	98.5-102.6	WuH16	2016	6.31	4.01	3.90
LWRP	*qLWRPB08*	B08	AhSNP4122007-AhSNP4048068	80.31	78.8-81.9	YangL16	2016	4.93	0.08	2.88
	qLWRPB10.1	B10	AhSNP669219-AhSNP214780	54.71	53.8-55.4	WuH16	2016	4.99	0.06	2.89
	qLWRPB10.2	B10	AhSNP353647-AhSNP310250	82.01	81-84.3	WuH16	2016	4.28	-0.06	2.60
	qLWRPB10.3	B10	AhSNP526110-AhSNP633440	88.61	87.2-93.6	WuH16	2016	4.18	-0.05	2.54
LWRS	*qLWRSB02.1*	B02	AhSNP13769754-AhSNP13888121	8.01	7-9.2	WuH15	2015	4.50	-0.05	2.59
	qLWRSB02.2	B02	AhSNP13898835-AhSNP13936477	54.11	52-55.2	WuH15	2015	4.64	0.05	2.75
	qLWRSB06	B06	AhSNP14760776-AhSNP14701698	150.51	148.4-151.7	WuH16	2016	15.41	4.03	7.27
SNPP	*qSNPPB07*	B07	AhSNP7931358-AhSNP8321099	24.61	21.6-28.7	WuH16	2016	5.23	0.05	2.87
