# Supplementary figures and images for: Development of a High-Density Genetic Map Based on Specific Length Amplified Fragment Sequencing and Its Application in Quantitative Trait Loci Analysis for Yield-Related Traits in Cultivated Peanut
Source: Front Plant Sci. 2018 Jun 26;9:827. doi: 10.3389/fpls.2018.00827 (PMC6028809; doi:10.3389/fpls.2018.00827)

Supplementary Figure S1. Number of each marker segregation type on the linkage map of p

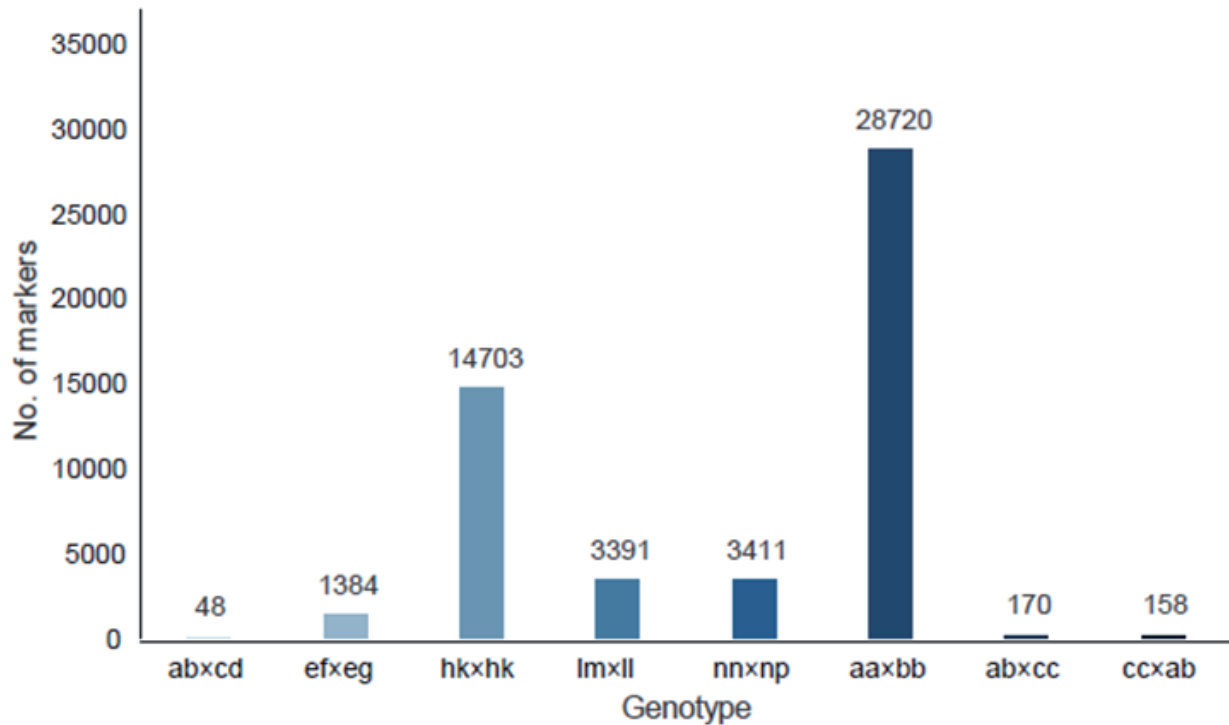

Supplement: Supplementary file 6 [file Image_1.PDF]

Supplementary Figure S6. The distribution of recombination rates along each chromosome.

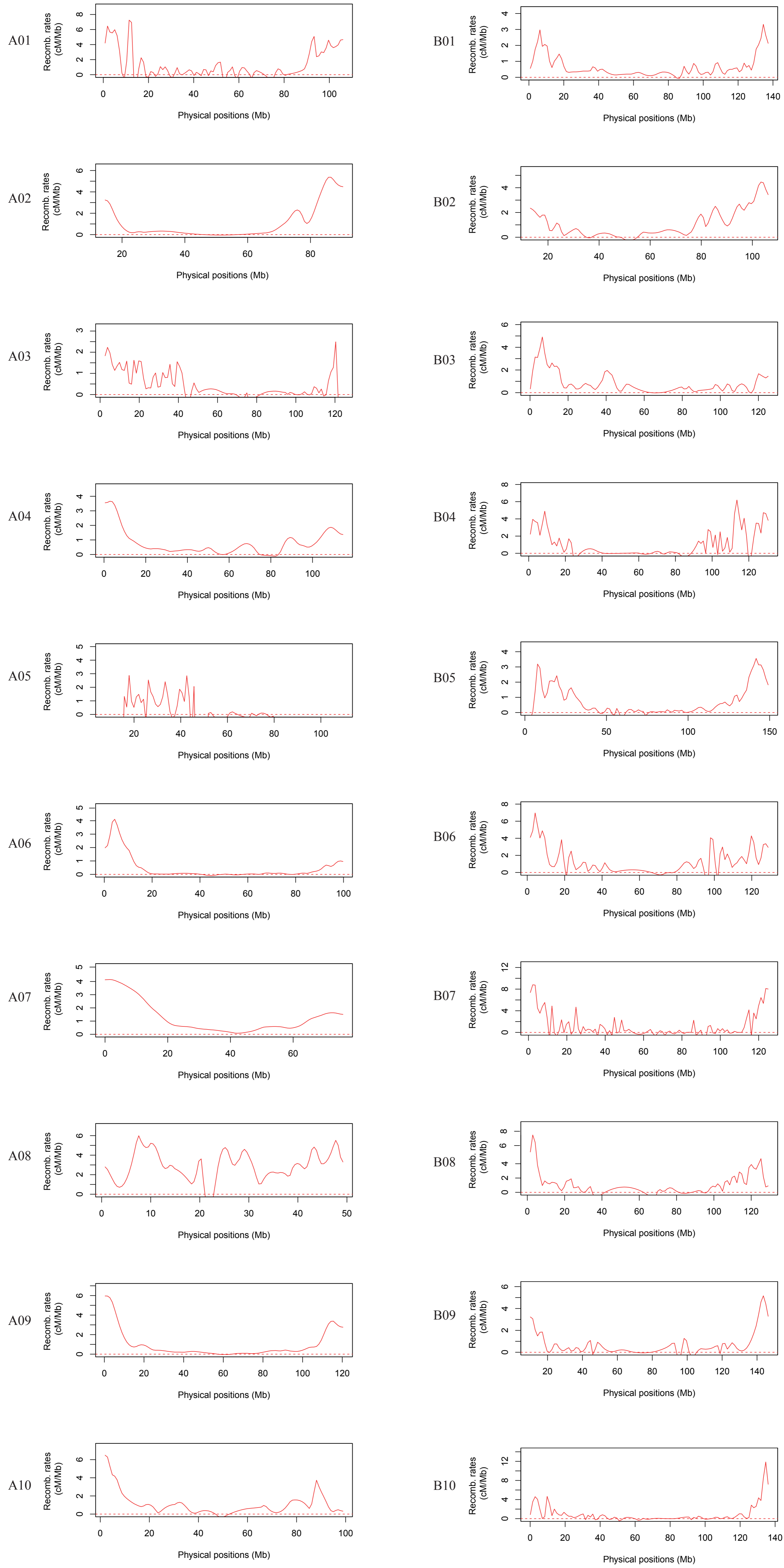

Supplement: Supplementary file 11 [file Image_6.PDF]
