# Supplementary material for: Development of a High-Density Genetic Map Based on Specific Length Amplified Fragment Sequencing and Its Application in Quantitative Trait Loci Analysis for Yield-Related Traits in Cultivated Peanut
Source: Front Plant Sci. 2018 Jun 26;9:827. doi: 10.3389/fpls.2018.00827 (PMC6028809; doi:10.3389/fpls.2018.00827)

Supplementary Figure S2. The SNP-based genetic linkage map for cultivated peanut using the 'ZH16' × 'sd-H1' population. SNP markers are preceded by 'AhSNP'. Markers are shown on right side of the LGs, while map distances are shown on the left side.

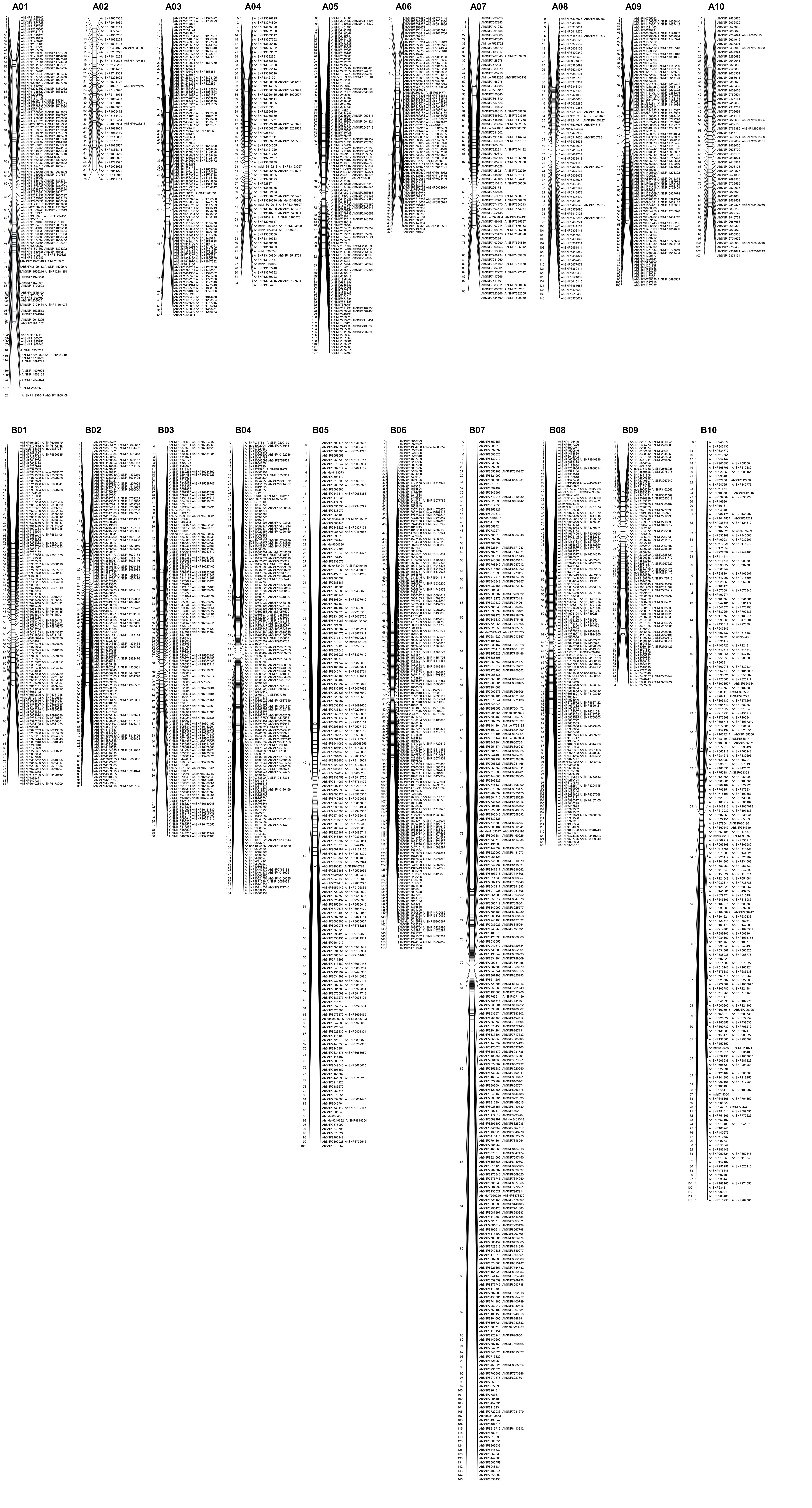

Supplement: Supplementary file 7 [file Image_2.PDF]
