# Supplementary material for: Development of a High-Density Genetic Map Based on Specific Length Amplified Fragment Sequencing and Its Application in Quantitative Trait Loci Analysis for Yield-Related Traits in Cultivated Peanut
Source: Front Plant Sci. 2018 Jun 26;9:827. doi: 10.3389/fpls.2018.00827 (PMC6028809; doi:10.3389/fpls.2018.00827)

Supplementary Figure S3. The type and composition of SNPs on the genetic map. (A) Percentages of diverse types of markers on each linkage group. The x-axis indicates the 20 linkage groups of the integrated map, the y-axis indicates the percentages of three types of markers: 'SNP_only,' 'InDel_only,' and SNP & InDel on each linkage group; (B) The number of different SNP mutation type on each linkage group; (C) The percentage of different SNP mutation type. R (G/A) and Y (T/C) are transition-type SNPs, and S(G/C), M(A/C), K(G/T) and W(A/T) are transversion-type SNPs.

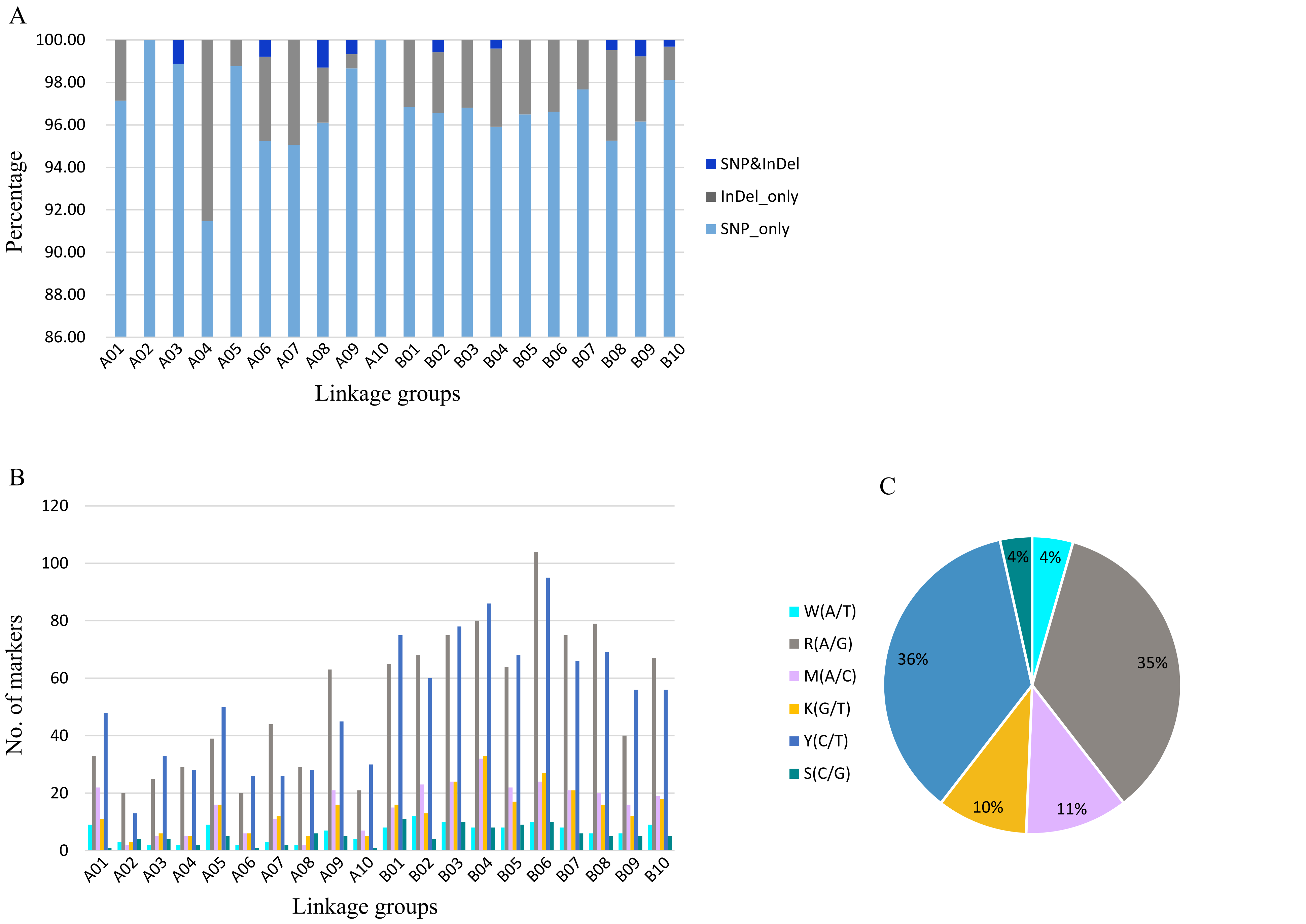

Supplement: Supplementary file 8 [file Image_3.PDF]
