# Supplementary material for: Development of a High-Density Genetic Map Based on Specific Length Amplified Fragment Sequencing and Its Application in Quantitative Trait Loci Analysis for Yield-Related Traits in Cultivated Peanut
Source: Front Plant Sci. 2018 Jun 26;9:827. doi: 10.3389/fpls.2018.00827 (PMC6028809; doi:10.3389/fpls.2018.00827)

Supplementary Figure S4. The segregation distortion regions (SDRs) on each linkage group. The distorted segregation markers were shown in red and the SDRs were defined on the right.

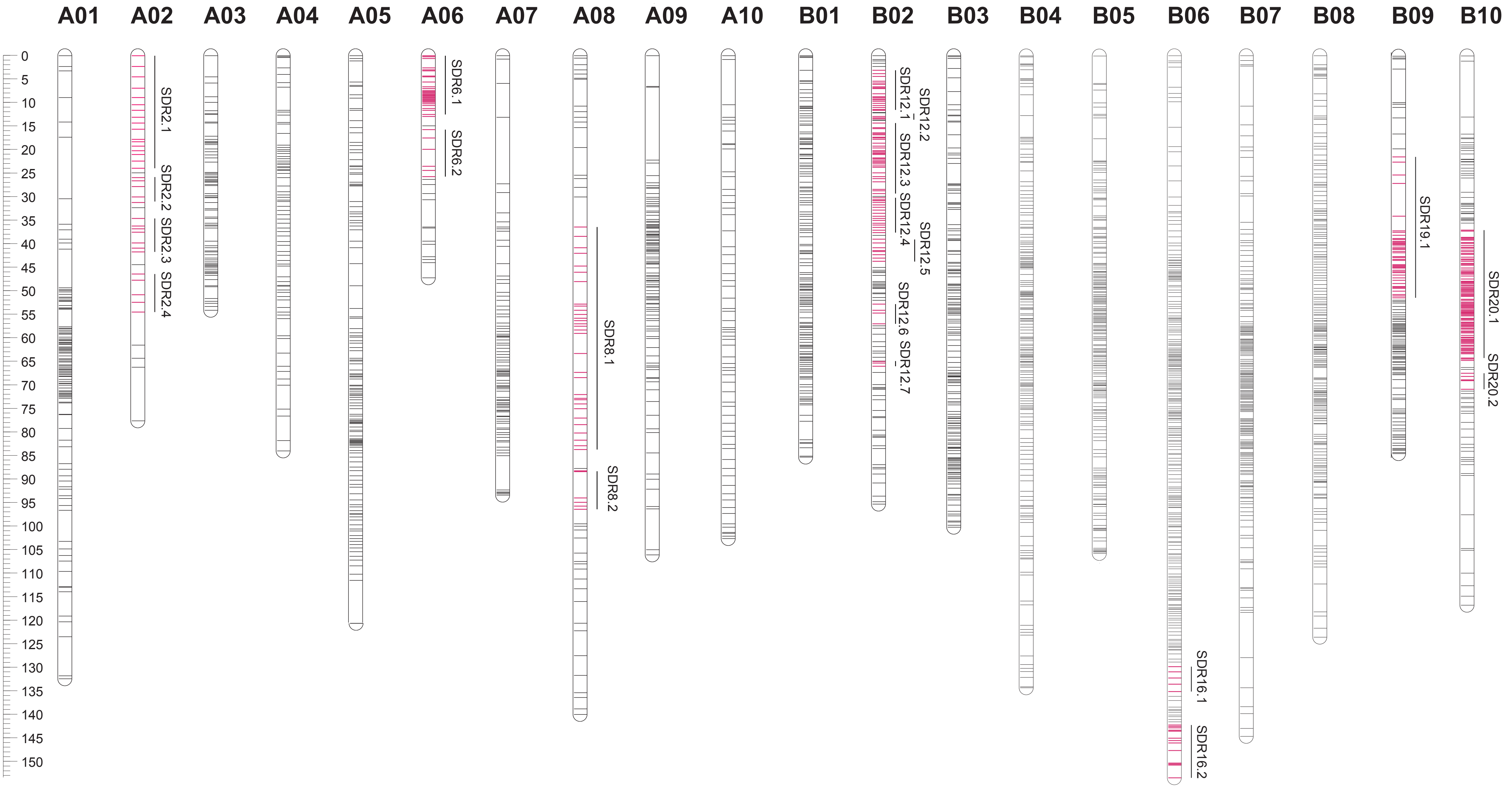

Supplement: Supplementary file 9 [file Image_4.PDF]
