# Supplementary material for: Development of a High-Density Genetic Map Based on Specific Length Amplified Fragment Sequencing and Its Application in Quantitative Trait Loci Analysis for Yield-Related Traits in Cultivated Peanut
Source: Front Plant Sci. 2018 Jun 26;9:827. doi: 10.3389/fpls.2018.00827 (PMC6028809; doi:10.3389/fpls.2018.00827)

Supplementary Figure S5. Haplotype map of the genetic map. Green represents male parent ‘sd-H1’, blue represents female parent ‘Zh16’, gray represents missing data, and red indicates heterozygosity. Each two columns represent the genotype of an individual. Rows correspond to genetic markers.

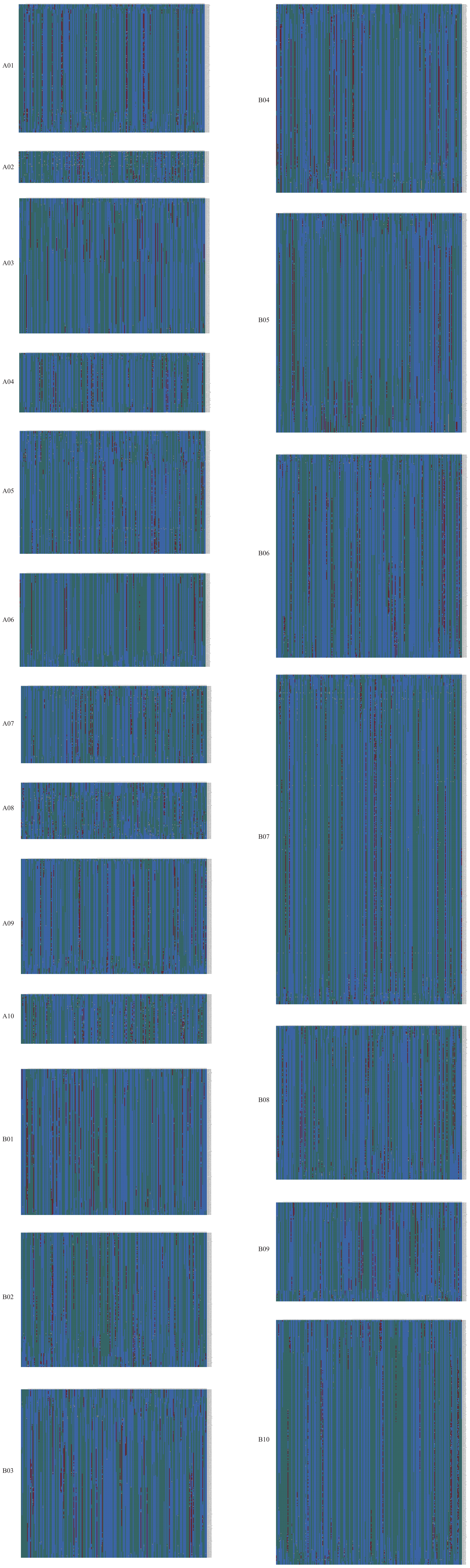

Supplement: Supplementary file 10 [file Image_5.PDF]
